# Accuracy of history, physical examination, cardiac biomarkers, and biochemical variables in identifying dogs with stage B2 degenerative mitral valve disease

**DOI:** 10.1111/jvim.16083

**Published:** 2021-03-01

**Authors:** Jenny Wilshaw, Steven L. Rosenthal, Gerhard Wess, Dave Dickson, Luca Bevilacqua, Emily Dutton, Michael Deinert, Ricardo Abrantes, Ingo Schneider, Mark A. Oyama, Sonya G. Gordon, Jonathan Elliott, Dong Xia, Adrian Boswood

**Affiliations:** ^1^ Department of Clinical Science and Services, Royal Veterinary College University of London London UK; ^2^ CVCA Cardiac Care for Pets Towson Maryland USA; ^3^ Clinic of Small Animal Medicine Ludwig‐Maximilians‐University of Munich Munich Germany; ^4^ HeartVets Porthcawl UK; ^5^ Stamford Veterinary Centre Lincolnshire UK; ^6^ Cheshire Cardiology Cheshire UK; ^7^ Fachtierarztpraxis Am Sandpfad Wiesloch Germany; ^8^ RA Kardiologie Muehlheim am Main Germany; ^9^ Tierarzt Ingo Schneider Nidderau Germany; ^10^ Department of Veterinary Clinical Studies, School of Veterinary Medicine University of Pennsylvania Philadelphia Pennsylvania USA; ^11^ College of Veterinary Medicine Texas A&M University College Station Texas USA; ^12^ Department of Comparative Biomedical Science, Royal Veterinary College University of London London UK; ^13^ Research Support Office, Royal Veterinary College University of London London UK

**Keywords:** canine, cardiac biomarker, machine learning, NT‐proBNP, prediction

## Abstract

**Background:**

Treatment is indicated in dogs with preclinical degenerative mitral valve disease (DMVD) and cardiomegaly (stage B2). This is best diagnosed using echocardiography; however, relying upon this limits access to accurate diagnosis.

**Objectives:**

To evaluate whether cardiac biomarker concentrations can be used alongside other clinical data to identify stage B2 dogs.

**Animals:**

Client‐owned dogs (n = 1887) with preclinical DMVD prospectively sampled in Germany, the United Kingdom, and the United States.

**Methods:**

Dogs that met inclusion criteria and were not receiving pimobendan (n = 1245) were used for model development. Explanatory (multivariable logistic regression) and predictive models were developed using clinical observations, biochemistry, and cardiac biomarker concentrations, with echocardiographically confirmed stage B2 disease as the outcome. Receiver operating characteristic curves assessed the ability to identify stage B2 dogs.

**Results:**

Age, appetite, serum alanine aminotransferase activity, body condition, serum creatinine concentration, murmur intensity, and plasma N‐terminal propeptide of B‐type natriuretic peptide (NT‐proBNP) concentration were independently associated with the likelihood of being stage B2. The discriminatory ability of this explanatory model (area under curve [AUC], 0.84; 95% confidence interval [CI], 0.82‐0.87) was superior to NT‐proBNP (AUC, 0.77; 95% CI, 0.74‐0.80) or the vertebral heart score alone (AUC, 0.76; 95% CI, 0.69‐0.83). A predictive logistic regression model could identify the probability of being stage B2 (AUC test set, 0.86; 95% CI, 0.81‐0.91).

**Conclusion and Clinical Importance:**

Our findings indicate accessible measurements could be used to screen dogs with preclinical DMVD. Encouraging at‐risk dogs to seek further evaluation could result in a greater proportion of cases being appropriately managed.

**Abbreviations**
ALKP: alkaline phosphatase
ALT: alanine aminotransferase
AUC: area under the curve
BCS: body condition score
BUN: blood urea nitrogen
CHF: congestive heart failure
CI: confidence interval
CKCS: Cavalier King Charles Spaniel
cTnI: cardiac troponin I
DMVD: degenerative mitral valve disease
EPIC: effect of pimobendan in dogs with preclinical myxomatous mitral valve disease and cardiomegaly
GBM: gradient boosting machine
GGT: gamma‐glutamyl transferase
LA:Ao: left atrial to aortic root ratio
LQ: lower quartile, 25th percentile
LVIDD: left ventricular internal diameter at end diastole
LVIDDN: left ventricular internal diameter at end diastole normalized to bodyweight (kg)
NT‐proBNP: N‐terminal propeptide of B‐type natriuretic peptide
OR: odds ratio
RAAS: renin angiotensin aldosterone system
SDMA: symmetric dimethylarginine
SVM: support vector machine
UQ: upper quartile, 75th percentile
VHS: vertebral heart score

## 1 INTRODUCTION

Degenerative mitral valve disease (DMVD) is an acquired condition characterized by progressive myxomatous degeneration of the mitral valve. As the most prevalent cardiac disease of the adult dog,^1^ it is estimated to affect 3.5% of dogs seen in primary‐care practice.^2^ Many dogs with DMVD experience a long, preclinical period, during which they might develop eccentric hypertrophy of the left‐sided chambers of the heart to compensate for chronic volume overload. These structural changes are used to identify dogs with more advanced preclinical disease in a staging scheme produced by the American College of Veterinary Internal Medicine.^3^ Dogs are classified as being in stage B2 if echocardiographic measurements of left atrial and left ventricular size exceed thresholds that are considered sufficiently indicative of cardiomegaly to inform decisions on case management. Preclinical dogs not meeting these echocardiographic criteria are classed as being in stage B1. Correctly identifying which preclinical dogs are in stage B2 is clinically important as the effect of pimobendan in dogs with preclinical myxomatous mitral valve disease and cardiomegaly (EPIC) study demonstrated a clear benefit to medically managing these cases.^4^ In the EPIC study, treatment with pimobendan reduced the hazard of reaching the study's endpoint of congestive heart failure (CHF), cardiac related death or euthanasia by approximately one third. Given the average life expectancy of a dog, prolongation of the preclinical phase of DMVD represents a marked extension of good quality life.

It is challenging to recognize whether a dog is in stage B2 using information obtained from an external examination. Clinical signs such as a cough, exercise intolerance, inappetence, and weight loss can occur with progression of preclinical disease but might be attributed to diseases of other systems because of their nonspecific nature.^5^, ^6^, ^7^, ^8^ Some of these findings, along with findings from physical examination, are associated with the severity of preclinical disease, however their ability to detect cardiomegaly has not been rigorously assessed. Instead, severity is most commonly evaluated using imaging studies. When performed by an experienced practitioner, echocardiography is considered the best method of staging preclinical disease.^3^ Factors related to the dog, owner, and primary‐care practice can influence the decision to pursue echocardiography,^9^, ^10^ so ultimately only a minority of cases might undergo advanced evaluation. For these reasons, there has been increasing interest in cardiac biomarkers, which could be used to evaluate a wider range of cases because of the relative simplicity of blood sampling. Research in dogs has predominantly focused on N‐terminal propeptide of B‐type natriuretic peptide (NT‐proBNP) and cardiac troponin I (cTnI) as their concentrations positively correlate with echocardiographic dimensions.^11^, ^12^, ^13^, ^14^, ^15^ There is value in interpreting these biomarkers alongside other risk factors for DMVD,^5^, ^16^, ^17^ so it is possible that a similar approach could be used to stage preclinical disease in cases where echocardiography is not available.

This study aimed to determine whether data from readily accessible tests could reliably differentiate between dogs in stages B1 and B2. It was hypothesized that dogs in stage B2 would display differences in these variables, consistent with having more advanced disease. Our objectives were to:

- Identify variables associated with the likelihood of being in stage B2,
- Determine whether analyzing several of these variables in combination was beneficial when making this distinction, and
- Develop a model capable of predicting an individual's risk of being in stage B2.

## 2 METHODS

This was a prospective, cross‐sectional study. Recruitment was international (January 2018‐June 2019), involving 18 centers in Germany, 25 in the United Kingdom, and 16 in the United States. Examinations were conducted by veterinary cardiologists with at least 1 of the following qualifications: a diploma of the cardiology subspecialty of the European or American College of Veterinary Internal Medicine; Royal College of Veterinary Surgeons cardiology diploma or certificate; membership of the Collegium Cardiologicum; or membership of the working cardiology group of the Deutsche Gesellschaft für Kleintiermedizin ‐ Deutsche Veterinärmedizinische Gesellschaft. The participation of residents‐in‐training was permitted if directly supervised by a cardiologist. The collection and storage of data were performed with owner consent and the approval of the Ethics and Welfare Committee of the Royal Veterinary College (URN: 2017 1749‐3).

### 2.1 Case selection

The study sample consisted of client‐owned dogs that received an echocardiographic diagnosis of DMVD after undergoing evaluation by a cardiologist; this was defined as visible prolapse or thickening of the mitral valve and associated apparatus, in combination with mitral regurgitation on color Doppler examination. Dogs were required to be ≥6 years old, weigh ≥2 and ≤25 kg, and have a left apical systolic murmur with a point of maximum intensity over the mitral valve. Dogs were excluded if they had radiographic, historical, or physical examination findings consistent with CHF or if they were already receiving treatment with a loop diuretic. Known comorbidities expected to interfere with echocardiographic measurements or biomarker concentrations were considered additional reasons for exclusion. These included endocrine disorders, renal disease/injury, conditions accompanied by marked systemic inflammation, marked hepatic disease and, with the exception of concurrent tricuspid regurgitation, cardiac disease other than DMVD. Dogs receiving treatment with corticosteroids were not excluded.

### 2.2 Study samples evaluated

**FIGURE 1:**
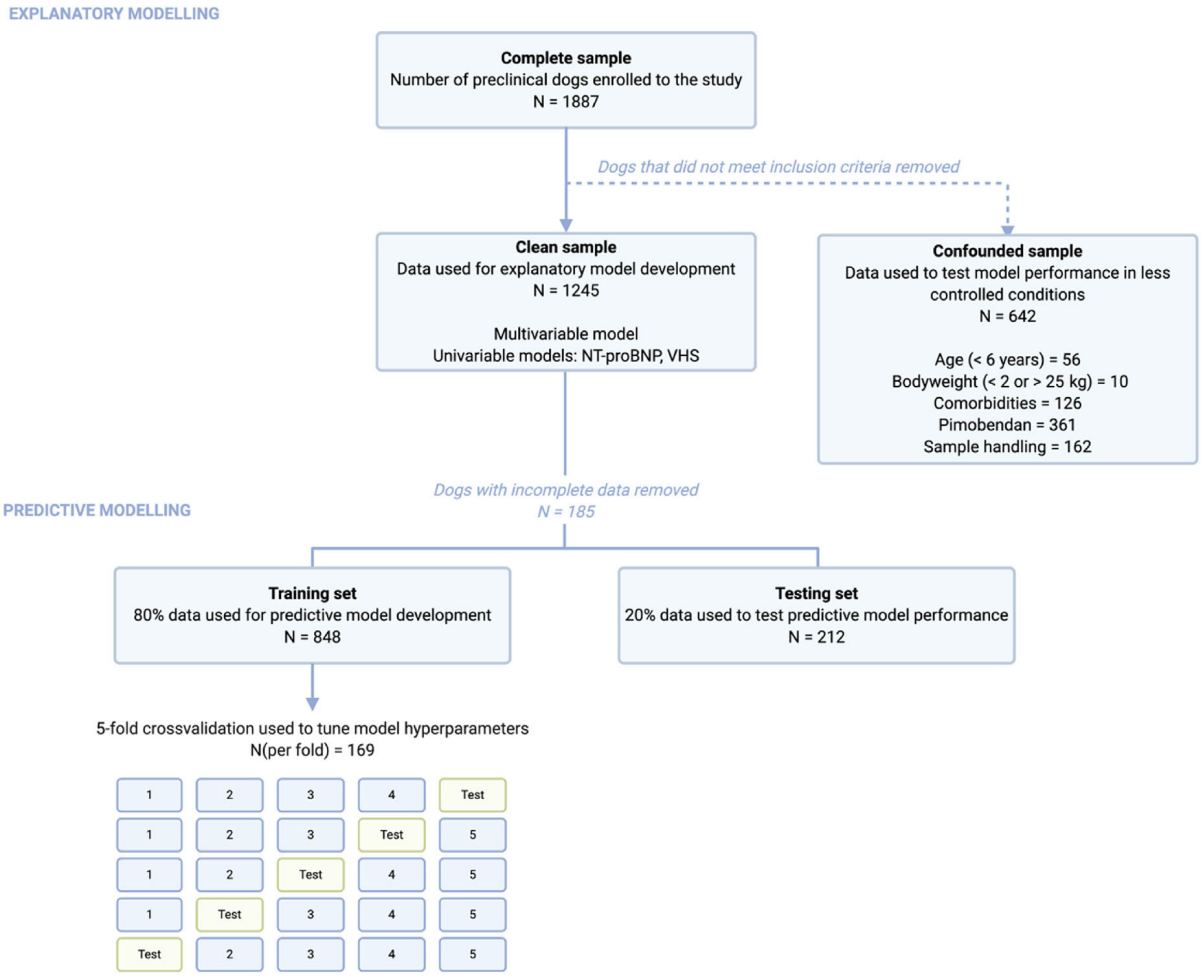
A flow chart indicating how data were partitioned before their inclusion in analyses

The presence of CHF and administration of loop diuretics excluded dogs from the study sample and any analyses conducted. From amongst the remaining “complete” sample, a “clean” sample was created to remove the influence of potential confounders from analyses. Dogs found to violate selection criteria, such as those with azotaemia, hypercalcaemia, endocrinopathies, or elevations in alanine aminotransferase activity (ALT, >4 × upper reference limit), were excluded from the “clean” sample,^18^ as well as dogs whose samples had taken longer than 72 hours to arrive at the reference laboratory. Dogs receiving treatment with pimobendan were excluded from the “clean” sample to eliminate the drug's influence on echocardiographic dimensions.^6^ The data from dogs excluded for all reasons other than CHF and the receipt of loop diuretics were retained and used to form a “confounded” sample for use in a subanalysis. This is summarized in Figure 1.

### 2.3 Clinical evaluation

Data were captured by veterinary cardiologists at the point of examination. It was noted if, in the past 6 months, the dog had developed a cough, exercise intolerance or a reduced appetite. Heart and respiratory rates were measured and the predominant heart rhythm throughout auscultation classified as sinus rhythm, sinus arrhythmia or “other.” Murmur intensity was initially attributed a value of I‐VI using a modified version of the Levine grading system (Supplementary Materials: Study Protocol); however, this was later reclassified to reduce complexity and account for the low occurrence of murmurs at either extreme of the grading system.^19^ Grade I and II murmurs were labeled as soft, grade III as moderate, grade IV as loud, and grades V and VI as thrilling.^20^ Body condition (BCS) was scored using the 9‐point scale.^21^ Echocardiography was used to obtain standard right parasternal views. Left atrial to aortic root ratio (LA:Ao) was recorded from a short‐axis, 2D view in early ventricular diastole.^22^ The left ventricular internal diameter in diastole (LVIDD) was recorded from a short‐axis, M‐mode view at the level of the chordae tendinae. Left ventricular internal diameters were normalized to bodyweight (LVIDDN) using the formula: LVIDDN = LVIDD (cm)/weight^0.294^ (kg).^23^ Stage B2 was defined as an LA:Ao ≥1.6 and LVIDDN ≥1.7.^3^ Dogs that did not meet both of these criteria were classified as stage B1. Where available, vertebral heart score (VHS) was recorded.^24^, ^25^

A venous blood sample was taken from all dogs to obtain serum biochemistry and cardiac biomarker concentrations. Processed aliquots were sent on ice to a research laboratory in Germany or the USA depending on the sample's point of origin (IDEXX BioResearch, Ludwigsburg, Germany; IDEXX BioAnalytics, California). For all dogs, NT‐proBNP concentrations were measured using the second‐generation ELISA: Canine Cardiopet proBNP, and cTnI concentrations were measured using a sandwich immunoassay: Advia Centaur TnI‐Ultra, Siemens Healthcare Diagnostics. Biochemistry assays used standard diagnostic methods on automated analyzers. Plasma NT‐proBNP concentrations and serum biochemistry profiles were produced at the time of sample receipt. Serum samples for measurement of cTnI were stored at −80°C and batch processed once recruitment had been completed. Results that fell below detection limits of assays were assigned the value of the lower limit.^5^, ^16^ All measurements underwent statistical analysis in Systéme Internationale units.

### 2.4 Analytical methods

Analyses were performed using commercial software and open‐source freeware (Python Software Foundation, Python Language Reference, v3.7; R v1.2.5019, R Foundation for Statistical Computing, Vienna, Austria; SPSS v26.0 for Macintosh, SPSS Inc, San Diego, California). Statistical significance was set as *P* < .05. Continuous data are reported as the median (lower quartile [LQ], upper quartile [UQ]) and categorical variables are presented as the proportion (frequency). The normality of continuous variables was assessed using histograms. Variables displaying a marked right skew were logarithmically transformed. Collinearity between variables was considered if Spearman's rho exceeded 0.7 or if the variance inflation factor was ≥5.^26^ Categorical variables with small group sizes were reclassified into broader levels before analyses.

#### 2.4.1 Factors associated with stage B2 DMVD

Within the “clean” sample, binary logistic regression was used to identify factors associated with the likelihood of having stage B2 disease. Cases were dichotomized according to whether or not they were in stage B2 and clinical data and blood test concentrations were entered as explanatory variables. Laboratory location was tested as a potential confounder. Univariable restricted cubic spline models were used to assess the assumption of linearity with the logit.^27^ When this was violated, continuous variables were quartile transformed for analysis. Variables that displayed an association at a univariable level (*P* < .2) were selected for inclusion in a multivariable analysis. Using complete data, backward stepwise elimination was used to select a preliminary main effects model based on likelihood ratio tests (*P* < .05) and the standardized change‐in‐estimate criterion (threshold = 20%, R package “abe” v3.0.1).^28^, ^29^ Variables excluded by univariable testing were then individually entered into the main effects model and retained if they induced a substantial change in coefficients (>20%) indicative of a confounding effect.^27^ Two‐way interaction terms were tested for variables remaining in the model as main effects and included in the final model if they displayed a significant association with disease stage.^30^ Estimated marginal means were calculated for all categorical variables that remained in the final explanatory model. To calculate estimated marginal means, continuous variables in the model were held at their mean and categorical variables were held at their reference group. Results would therefore reflect changes in the likelihood of being stage B2 across different categories of the variable being examined. Overall model fit was assessed using the Hosmer‐Lemeshow test before model acceptance. Results are reported as coefficients (β) and odds ratios (ORs) with 95% confidence intervals (CIs).

#### 2.4.2 Discriminatory ability in alternate settings

Model performance was assessed by plotting a receiver operating characteristic curve using predicted probabilities and calculating the area under the curve (AUC) with 95% CIs. In order to evaluate the degree to which comorbidities, sample handling, or pimobendan administration affected discriminatory ability, the coefficients for the explanatory multivariable model were applied to data from the “complete” and “confounded” samples. Respective AUCs were compared to the “clean” sample using a DeLong test.^31^ The discriminatory ability of the explanatory multivariable model was additionally compared to other methods that could be used to identify stage B2 DMVD. Disease stage was regressed on NT‐proBNP alone and VHS alone, from which AUC was calculated.

#### 2.4.3 Predicting preclinical disease stage

A series of models were developed to evaluate whether preclinical disease status could be predicted in a subset of known data. Binary logistic regression was tested alongside more complex machine learning (ML) algorithms which do not rely on the same assumptions as regression and offer protection against overfitting.^32^ These were ridge regression,^33^ support vector machines (SVM),^34^ random forest,^35^ and the gradient boosting machine (GBM) XGBoost.^36^ The clean data were partitioned with 80% used to train models and 20% used to test performance (Figure 1). Rows containing missing data were not included in this split. Data were then preprocessed according to the requirements of each model (Supplementary Methods). In multivariable logistic regression, features underwent univariable screening (*P* < .2) and then backward stepwise elimination with the residual chi‐squared as the stopping criterion (*P* < .05) to select a model based on parsimony.^37^ The hyperparameters of ML models were tuned using a grid search in a 5‐fold cross validation loop of the training set. Fitted models were applied to the test set to generate predicted probabilities of being stage B2 and assess performance. Plots of variable importance were also produced where possible for ML models.

## 3 RESULTS

The complete study sample consisted of 1887 dogs with preclinical DMVD and the median number of dogs recruited by each center was 24 (range, 1‐116). Six hundred forty‐two dogs were excluded from the “clean” sample on the basis of having 1 or more of: age <6 years old (n = 56, 2.75%), bodyweight <2 or >25 kg (n = 10, 0.53%), comorbidities (n = 126, 6.68%), pimobendan medication (n = 361, 19.13%), or inappropriate sample handling (n = 162, 8.59%). Of the 361 dogs receiving treatment with pimobendan, only 56.51% (n = 204) met the criteria for stage B2 disease. The resulting “clean” sample comprised 1245 dogs (Figure 1).

Among the “clean” sample, 27.1% (n = 337) of dogs were classified as having stage B2 disease. The most common breed evaluated was the Cavalier King Charles Spaniel (n = 292, 27.07%), followed by Chihuahuas (n = 84, 6.74%), Jack Russell Terriers (n = 56, 4.50%), Shih Tzus (n = 43, 3.45%), and Cocker Spaniels (n = 43, 3.45%). The median value of NT‐proBNP in stage B1 was 589.50 pmol/L (LQ, 373.50; UQ, 877.25) and 1188.00 pmol/L in stage B2 (LQ, 774.00; UQ, 2000.00) (Supplementary Figure 1). Thirty‐one percent of dogs (n = 387) reported clinical signs, of which a cough was the most common complaint (n = 299, 24.02%). Only 14.1% dogs (n = 175) had a VHS reported, with the median score being 11.00 (LQ, 10.50; UQ, 11.50). Additional descriptive statistics are reported in Tables 1 and 2; and Supplementary Tables 1 and 2.

**TABLE 1:** Characteristics of dogs with stage B1 or stage B2 degenerative mitral valve disease

Variable		B1 (n = 908)	B2 (n = 337)
Age (years)		10.00 (8.00, 11.67)	10.00 (8.50, 11.35)
BCS	≤3	1.76% (16)	3.86% (13)
	*1*	0% (0)	0% (0)
	*2*	0.22% (2)	0% (0)
	*3*	1.54% (14)	3.86% (13)
	4	16.63% (151)	14.24% (48)
	5	41.63% (378)	43.32% (146)
	6	22.36% (203)	28.49% (96)
	7	12.78% (116)	6.82% (23)
	≥8	4.41% (40)	2.67% (9)
	*8*	3.85% (35)	2.37% (8)
	*9*	0.55% (5)	0.30% (1)
Breed	CKCS	22.69% (206)	25.52% (86)
Sex	Female entire	4.07% (37)	2.67% (9)
	Female neutered	39.10% (355)	37.39% (126)
	Male entire	12.11% (110)	11.28% (38)
	Male neutered	44.71% (406)	48.66% (164)
Weight (kg)		9.30 (6.80, 12.60)	8.70 (6.50, 11.30)

*Notes*: Descriptive statistics for the dogs in the clean sample. These are reported as the median (LQ, UQ) for continuous variables and the proportion (frequency) for categorical variables. N represents the number of dogs belonging to a group. The following variables contained missing data: age (B1: 0.11%, n = 1; B2: 0.30%, n = 1), BCS (B1: 0.44%, n = 4; B2: 0.59%, n = 2). BCS, body condition score; CKCS, Cavalier King Charles Spaniel.

**TABLE 2:** Clinicopathological data for dogs with stage B1 or stage B2 degenerative mitral valve disease

Variable		B1 (n = 908)	B2 (n = 337)
Appetite	Decreased	0.66% (6)	2.67% (9)
Cardiac biomarkers	cTnI (ng/mL)	0.05 (0.03, 0.08)	0.06 (0.04, 0.10)
	NT‐proBNP (pmol/L)	589.50 (373.50, 877.25)	1188.00 (774.00, 2000.00)
Cardiac medications	ACEi	3.41% (31)	3.56% (12)
	Spironolactone	0.44% (4)	1.48% (5)
Cough	Yes	20.15% (183)	34.4% (116)
Exercise tolerance	Decreased	9.58% (87)	15.43% (52)
Heart rate		120.00 (104.00, 132.00)	128.00 (117.00, 140.00)
Heart rhythm	Regular rhythm	61.78% (561)	71.81% (242)
	Sinus arrhythmia	35.79% (325)	26.41% (89)
	Other	2.42% (22)	1.48% (5)
LA:Ao		1.38 (1.26, 1.52)	1.85 (1.72, 2.02)
LVIDDN (cm/kg^0.294^)		1.52 (1.47, 1.70)	1.93 (1.81, 2.11)
Murmur intensity	Soft (I‐II)	25.55% (232)	4.45% (15)
	*I*	2.75% (25)	0.30% (1)
	*II*	22.80% (207)	4.15% (14)
	Moderate (III)	44.16% (401)	25.82% (87)
	Loud (IV)	24.89% (226)	48.66% (164)
	Thrilling (V‐VI)	5.07% (46)	21.07% (71)
	*V*	3.96% (36)	19.29% (65)
	*VI*	1.10% (10)	1.78% (6)
Respiratory rate		26.00 (20.00, 32.00)	26.00 (22.00, 32.00)
Serum biochemistry	Albumin (g/L)	33.00 (31.00, 35.00)	33.00 (30.00, 35.00)
	ALKP (U/L)	52.50 (29.00, 125.00)	61.00 (33.00, 173.00)
	ALT (U/L)	48.00 (34.00, 76.00)	51.00 (37.00, 76.00)
	Bilirubin (μmol/L)	3.20 (2.40, 3.42)	3.10 (2.10, 3.42)
	BUN (mmol/L)	6.10 (4.80, 7.85)	5.90 (5.00, 7.50)
	Calcium (mmol/L)	2.50 (2.40, 2.60)	2.50 (2.40, 2.60)
	Chloride (mmol/L)	111.00 (109.00, 113.00)	111.00 (109.00, 113.00)
	Cholesterol (mmol/L)	6.28 (5.20, 7.50)	5.90 (4.86, 7.12)
	Creatinine (μmol/L)	65.00 (53.04, 79.56)	61.88 (52.00, 73.00)
	GGT (U/L)	4.00 (3.00, 5.50)	4.00 (3.00, 6.00)
	Globulin (g/L)	30.00 (28.00, 33.00)	30.00 (27.00, 33.00)
	Glucose (mmol/L)	5.30 (4.80, 5.71)	5.30 (4.90, 5.66)
	Phosphate (nmol/L)	1.20 (1.00, 1.40)	1.26 (1.10, 1.42)
	Potassium (mmol/L)	4.50 (4.30, 4.80)	4.60 (4.30, 4.90)
	SDMA (μg/dL)	10.00 (9.00, 13.00)	10.00 (9.00, 12.00)
	Sodium (mmol/L)	148.00 (147.00, 150.00)	148.00 (147.00, 150.00)
VHS (n = 175)		n = 97, 10.80 (10.25, 11.10)	n = 78, 11.50 (11.00, 12.25)

*Notes*: Descriptive statistics for the dogs in the clean sample. These are reported as the median (LQ, UQ) for continuous variables and the proportion (frequency) for categorical variables. N represents the number of dogs belonging to a group. The following variables contained missing data: cTnI (B1: 1.43%, n = 13; B2: 1.78%, n = 6), heart rate (B1: 0.22%, n = 2), heart rhythm (B2: 0.30%, n = 1), murmur intensity (B1: 0.33%, n = 3), respiratory rate (B1: 12.44%, n = 113; B2: 7.72%, n = 26), albumin (B2: 0.30%, n = 1), ALKP (B1: 0.11%, n = 1), bilirubin (B1: 0.22%, n = 2), BUN (B1: 0.11%, n = 1), calcium (B1: 0.33%, n = 3), cholesterol (B1: 0.22%, n = 2), GGT (B1: 0.11%, n = 1; B2: 0.59%, n = 2), glucose (B2: 0.59%, n = 2), phosphate (B1: 0.11%, n = 1), potassium (B1: 0.33%, n = 3), SDMA (B1: 1.10%, n = 10; B2: 0.59%, n = 2). ALKP, alkaline phosphatase; ALT, alanine aminotransferase; BUN, blood urea nitrogen; cTnI, cardiac troponin I; GGT, gamma‐glutamyl transferase; NT‐proBNP, N‐terminal propeptide of B‐type natriuretic peptide; SDMA, symmetric dimethylarginine; VHS, vertebral heart score.

### 3.1 Factors associated with stage B2 DMVD

**FIGURE 2:**
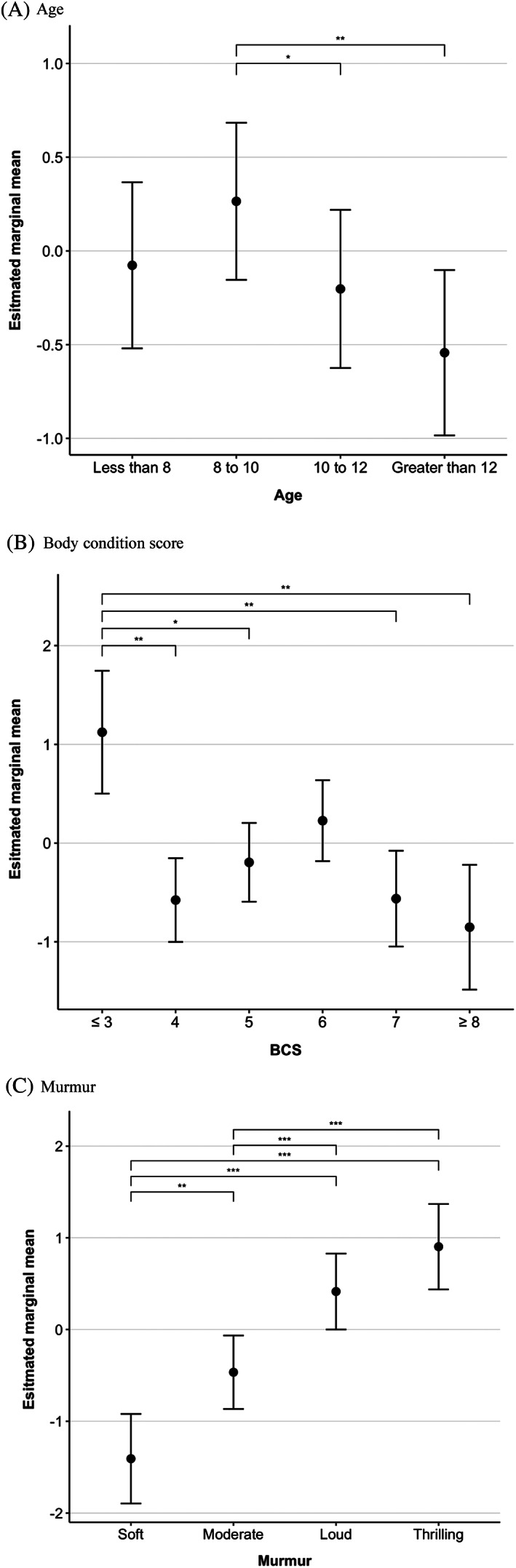
Estimated values of the log likelihood of being in stage B2 at different levels of categorical variables present in the explanatory multivariable model. Estimated marginal means were calculated when other variables in the explanatory multivariable logistic regression were held at a constant (continuous variables = mean, categorical variables = reference group). Estimated marginal means were compared using Fischer's Least Significant Difference test and significant pairwise comparisons are displayed using asterisks (*<.05, **<.01, ***<.001). Dots are used to indicate the estimated marginal mean and error bars are used to indicate the standard error of the mean

In univariable testing, age, log_10_(Bilirubin), and log_10_(cTnI) were nonlinearly related to the outcome, so were categorized into quartiles using their nontransformed values. Of the variables tested, 18 were associated with disease stage at a univariable level (Supplementary Table 3). In the multivariable analysis, the following variables were identified as independent risk factors: age, ALT, appetite, BCS, creatinine concentration, murmur intensity, and NT‐proBNP concentration (Table 3). A reduction in appetite and lower BCS were associated with greater odds of being in stage B2. Post hoc testing of BCS demonstrated that this was true when underweight scores (BCS ≤3) were compared to almost all other values (Table 4; Figure 2B). Estimated marginal means for murmur intensity showed that the likelihood of being in stage B2 was greater when murmurs were more audible, with the comparison between loud and thrilling murmurs being the only pairwise combination that did not significantly differ (Table 4; Figure 2C). Age was also associated with the outcome, with dogs between 8 and 10 years old at greatest risk. In dogs older than 10, the likelihood of being stage B2 was significantly lower (Table 4; Figure 4A). Increasing serum creatinine concentrations were associated with a reduction in the odds of being in stage B2 (β, −0.02; OR, 0.98; 95% CI, 0.97‐0.99; *P* < .001). In contrast, both log_10_(NT‐proBNP) and log_10_(ALT) were positively related to the odds of being in stage B2 when modeled as main effects. ALT and NT‐proBNP negatively interacted, meaning that the association between log_10_(NT‐proBNP) and the outcome was not as strong at higher values of log_10_(ALT), and the association for log_10_(ALT) was not as strong at high values of log_10_(NT‐proBNP) (Supplementary Figure 2). Appetite and creatinine also produced a significant interaction term; however, this was not included in the final model as there was potential for spurious results in the few dogs where appetite was reduced (1.15%, n = 12) (normal β, −0.02; OR, 0.98; 95% CI, 0.97‐0.99; *P* < .001; reduced β, −0.18; OR, 0.84; 95% CI, 0.72‐0.98; *P* < .03).

**TABLE 3:** The results of the explanatory multivariable logistic regression model showing risk factors associated with having stage B2 DMVD

Variable	β	Odds ratio (95% CIs)	*P*
Intercept	−23.91	4.07 e^−11^ (4.59 e^−16^ ‐ 3.81 e^−6^)	<.001
Age (≤8)	‐	‐	‐
Age (8‐10)	0.34	1.41 (0.89‐2.24)	.15
Age (10‐12)	−0.13	0.88 (0.54‐1.44)	.61
Age (>12)	−0.47	0.63 (0.36‐1.10)	.11
Appetite (Decreased)	2.38	10.85 (2.60‐53.31)	.002
Log_10_(ALT)	7.45	1773.22 (3.32‐7.99 e^5^)	.02
BCS (≤3)	‐	‐	‐
BCS (4)	−1.70	0.18 (0.06‐0.53)	.002
BCS (5)	−1.32	0.27 (0.10‐0.74)	.01
BCS (6)	−0.90	0.41 (0.15‐1.14)	.08
BCS (7)	−1.69	0.19 (0.06‐0.58)	.004
BCS (≥8)	−1.98	0.14 (0.03‐0.54)	.005
Creatinine	−0.02	0.98 (0.97‐0.99)	<.001
Murmur (soft)	‐	‐	‐
Murmur (moderate)	0.94	2.57 (1.38‐5.09)	.004
Murmur (loud)	1.82	6.19 (3.37‐12.21)	<.001
Murmur (thrilling)	2.31	10.10 (4.81‐22.32)	<.001
Log_10_(NT‐proBNP)	7.77	2374.40 (49.23‐1.12 e^5^)	<.001
Log_10_(ALT)*Log_10_(NT‐proBNP)	−2.25	0.10 (0.01‐0.89)	.04

*Notes*: For continuous variables, the odds ratio (OR) represents the change in odds associated with a 1‐unit increase in a variable according to its scale of measurement. This differs for logarithmically transformed variables where a 10‐fold change is used instead. For categorical variables, the OR represents the difference in odds when a category is compared to the reference group. Values of OR greater than 1 indicate that the odds of being in stage B2 increased with this variable. Values between 0 and 1 indicate that the odds reduced with this variable. Age was measured in years. ALT was measured in U/L. Creatinine underwent analysis in Systéme International (SI) units (μmol/L) so the coefficient reported reflects the change associated with a 1‐unit increase on this scale. ALT, alanine aminotransferase; BCS, body condition score; CI, confidence intervals; e, multiply by 10^x^; log_10_, logarithmic transformation to the base 10; NT‐proBNP, N‐terminal propeptide of B‐type natriuretic peptide; *P*, statistical significance; β, regression coefficient. A hyphen (‐) indicates that this category was used as a reference group for comparisons. The following levels of categorical variables were chosen as reference groups to provide a suitable point of comparison: age, ≤8 years; appetite, not reduced; BCS, ≤3; murmur intensity, soft.

**TABLE 4:** Pairwise comparisons of estimated marginal means for categorical variables in the multivariable model

Level 1	Level 2	Δβ (±SE)	*P*
Age			
Age (≤8)	Age (8‐10)	−0.34 (±0.24)	.15
Age (≤8)	Age (10‐12)	0.13 (±0.25)	.61
Age (≤8)	Age (>12)	0.47 (±0.29)	.11
Age (8‐10)	Age (10‐12)	0.47 (±0.22)	.03
Age (8‐10)	Age (>12)	0.81 (±0.26)	.002
Age (10‐12)	Age (>12)	0.34 (±0.26)	.19
Body condition score			
BCS (≤3)	BCS (4)	1.70 (±0.54)	.002
BCS (≤3)	BCS (5)	1.32 (±0.51)	.01
BCS (≤3)	BCS (6)	0.90 (±0.52)	.08
BCS (≤3)	BCS (7)	1.69 (±0.58)	.004
BCS (≤3)	BCS (≥8)	1.98 (±0.71)	.005
BCS (4)	BCS (5)	−0.38 (±0.25)	.12
BCS (4)	BCS (6)	−0.80 (±0.27)	.003
BCS (4)	BCS (7)	−0.01 (±0.37)	.97
BCS (4)	BCS (≥8)	0.28 (±0.54)	.61
BCS (5)	BCS (6)	−0.42 (±0.21)	.04
BCS (5)	BCS (7)	0.37 (±0.33)	.26
BCS (5)	BCS (≥8)	0.66 (±0.52)	.20
BCS (6)	BCS (7)	0.79 (±0.34)	.02
BCS (6)	BCS (≥8)	1.08 (±0.52)	.04
BCS (7)	BCS (≥8)	0.29 (±0.59)	.62
Murmur intensity			
Murmur (soft)	Murmur (moderate)	−0.94 (±0.33)	.004
Murmur (soft)	Murmur (loud)	−1.82 (±0.33)	<.001
Murmur (soft)	Murmur (thrilling)	−2.31 (±0.39)	<.001
Murmur (moderate)	Murmur (loud)	−0.88 (±0.19)	<.001
Murmur (moderate)	Murmur (thrilling)	−1.37 (±0.29)	<.001
Murmur (loud)	Murmur (thrilling)	−0.49 (±0.28)	.08

*Notes*: Results are presented for the change in coefficients (Δβ = β level 1 − β level 2) and significance of this pairwise comparison. Estimated marginal means were calculated with other variables held constant at the mean of continuous variables and reference group of categorical variables. Pairwise comparisons were performed using Fisher's Least Significant Difference (LSD) test. BCS, body condition score; Δβ, difference in coefficients; *P*, significance.

### 3.2 Discriminatory ability in alternate settings

**FIGURE 3:**
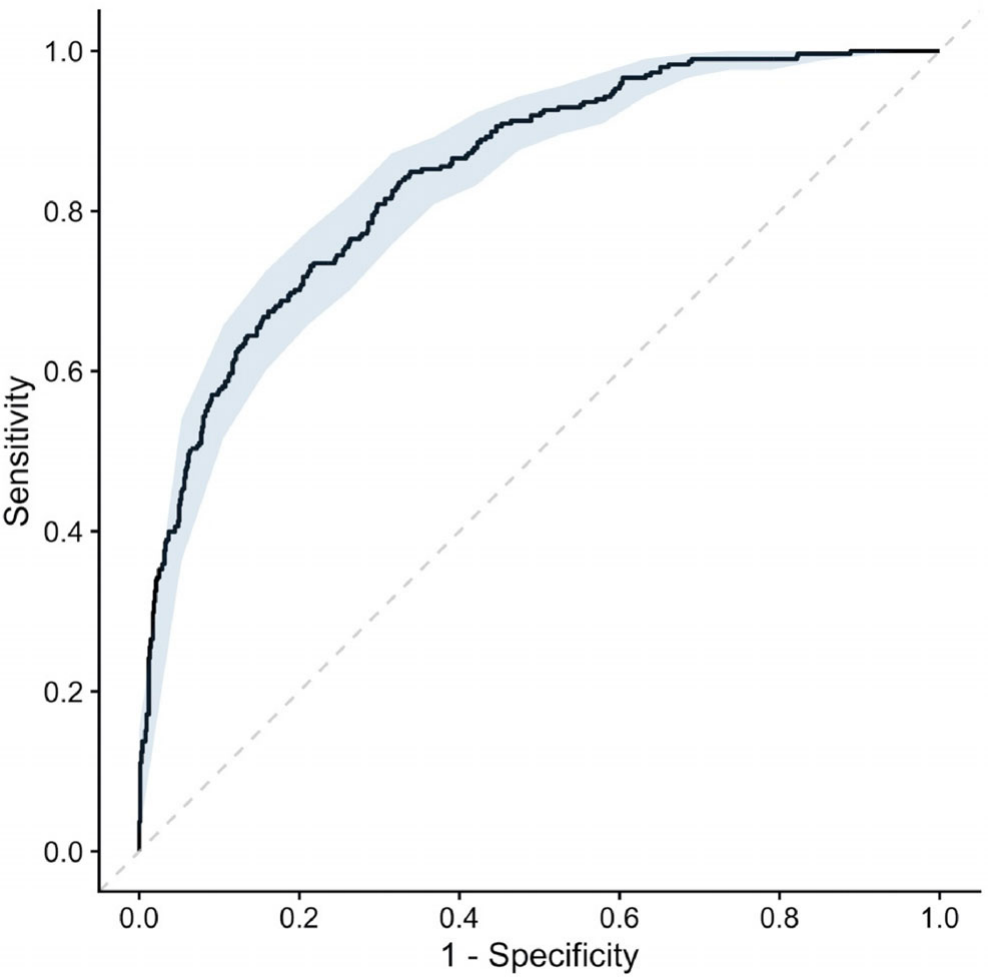
A receiver operating characteristic curve for the explanatory multivariable logistic regression analysis of risk factors associated with having stage B2 disease. 95% confidence intervals are represented by the blue area around the receiver operating curve. Area under the curve: 0.84 (0.82‐0.87)

**FIGURE 4:**
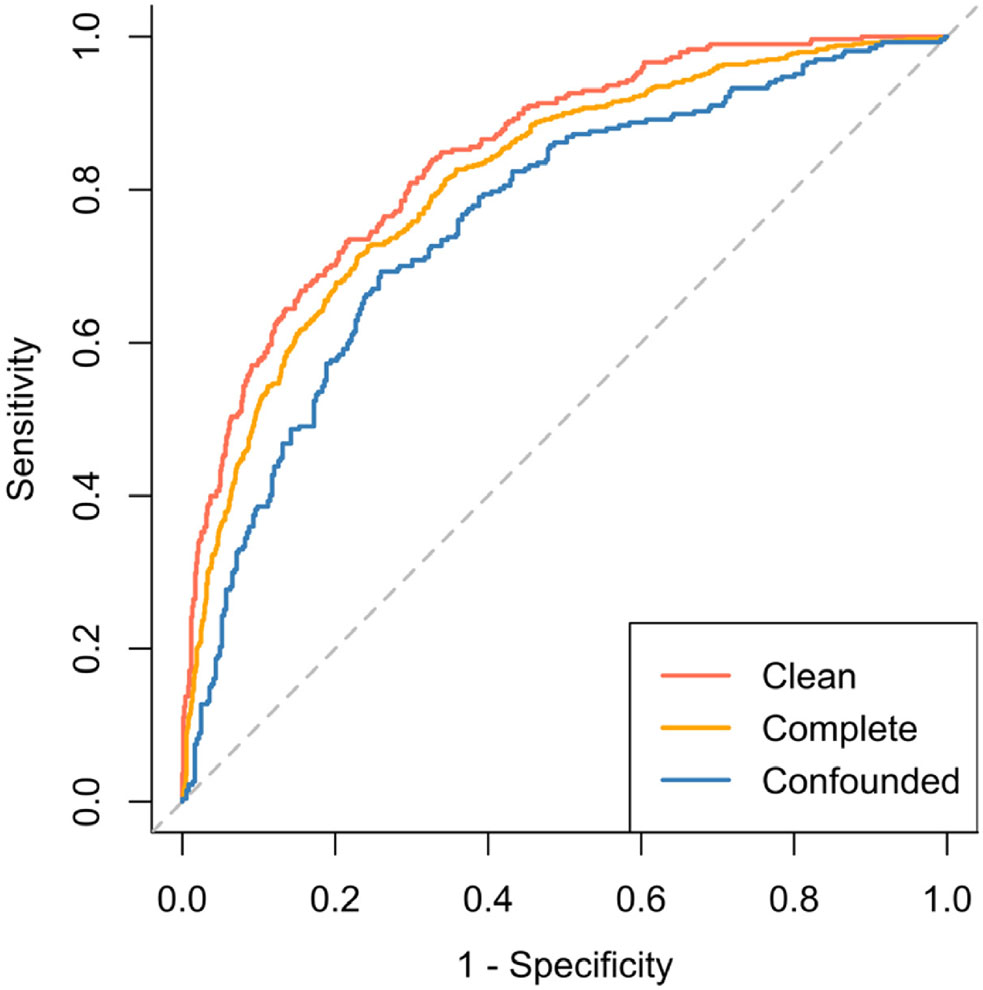
Discriminatory performance of the explanatory multivariable logistic regression model when applied to the clean, complete and confounded samples. The area under the receiver operating characteristic curves were as follows: for the clean sample 0.84 (0.82‐0.87); for the complete sample 0.81 (0.79‐0.83); for the confounded sample 0.76 (0.72‐0.80)

**FIGURE 5:**
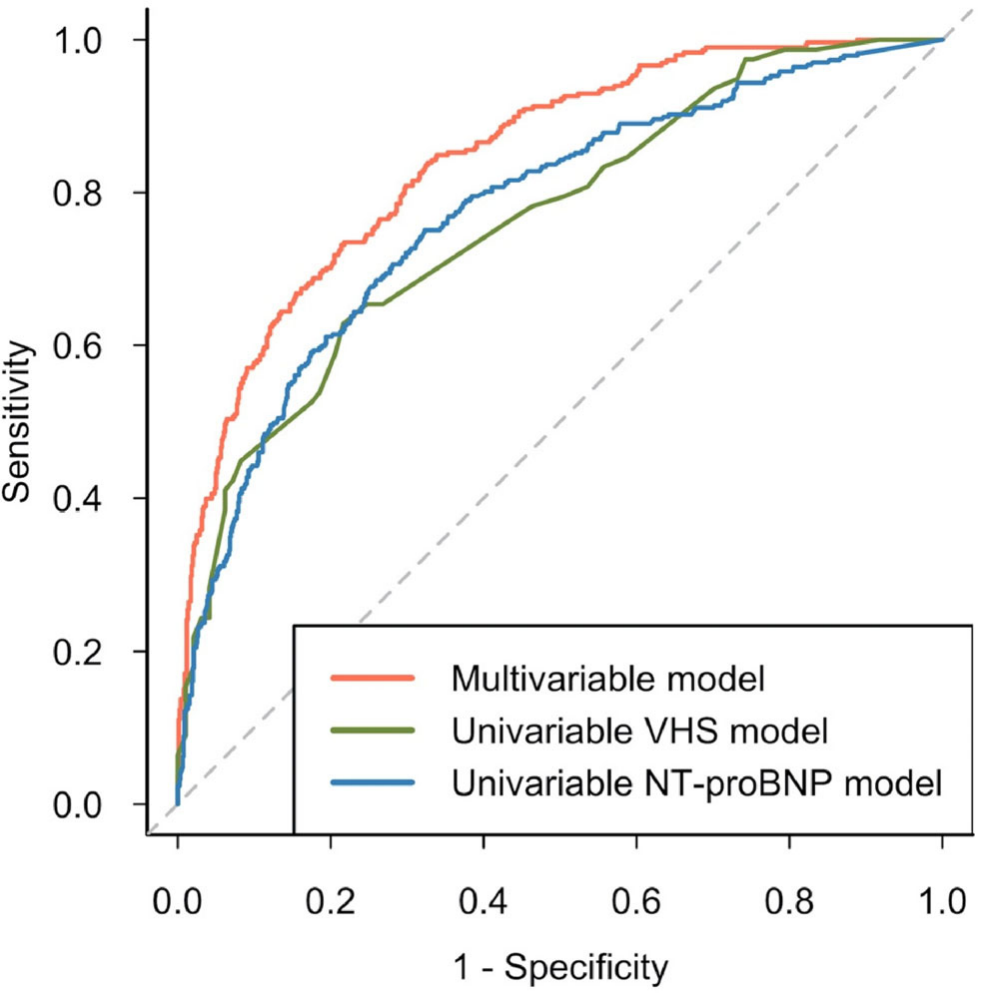
Discriminatory performance of the explanatory multivariable logistic regression model in comparison to using NT‐proBNP alone or the vertebral heart score. The area under the receiver operating characteristic curves were as follows: multivariable logistic regression model 0.84 (0.82‐0.87); univariable NT‐proBNP model 0.77 (0.74‐0.80); univariable vertebral heart score model 0.76 (0.69‐0.83). NT‐proBNP, N‐terminal propeptide of B‐type natriuretic peptide; VHS, vertebral heart score

The final explanatory multivariable model was shown to discriminate well between preclinical disease stages (AUC, 0.84; 95% CI, 0.82‐0.87; Nagelkerke R^2^, 0.42) (Figure 3). When applied to the “complete” sample, discriminatory performance decreased by a small amount (AUC, 0.81; 95% CI, 0.79‐0.83; *P* = .05 in comparison to the clean dataset) (Figure 4). In the “confounded” sample, comprised of data that had not been used for model derivation, performance was fair but significantly lower than when tested in more optimal conditions (AUC, 0.76; 95% CI, 0.72‐0.80; *P* < .001 in comparison to the clean dataset). Univariable models produced for NT‐proBNP and VHS found that both variables were positively associated with the odds of being in stage B2 (NT‐proBNP: β, 3.65; OR, 38.45; 95% CI, 23.14‐65.42; *P* < .001; Nagelkerke R^2^, 0.26; VHS: β, 1.28; OR, 3.61; 95% CI, 2.34‐5.96; *P* < .001; Nagelkerke R^2^, 0.29). As well as having smaller R^2^ values, AUCs for NT‐proBNP and VHS were lower and had wider CIs than the multivariable explanatory model, indicating that the multivariable model could better discriminate between stages B1 and B2 than either NT‐proBNP or VHS on their own (NT‐proBNP: AUC, 0.77; 95% CI, 0.74‐0.80; VHS: AUC, 0.76; 95% CI, 0.69‐0.83) (Figure 5). When compared, this difference in performance was significant (NT‐proBNP: *P* < .001; VHS: *P* = .03). If used in isolation to identify dogs as risk of being in stage B2, a cutoff value for NT‐proBNP of 1100 pmol/L had a sensitivity of 56%, specificity of 85%, positive predictive value (PPV) of 57%, and negative predictive value (NPV) of 84% with a pretest probability of 27%. A cutoff value for VHS of 11.5 had a sensitivity of 53%, specificity of 82%, PPV of 71%, and NPV of 68% with a pretest probability of 45%.

### 3.3 Predicting preclinical disease stage

**FIGURE 6:**
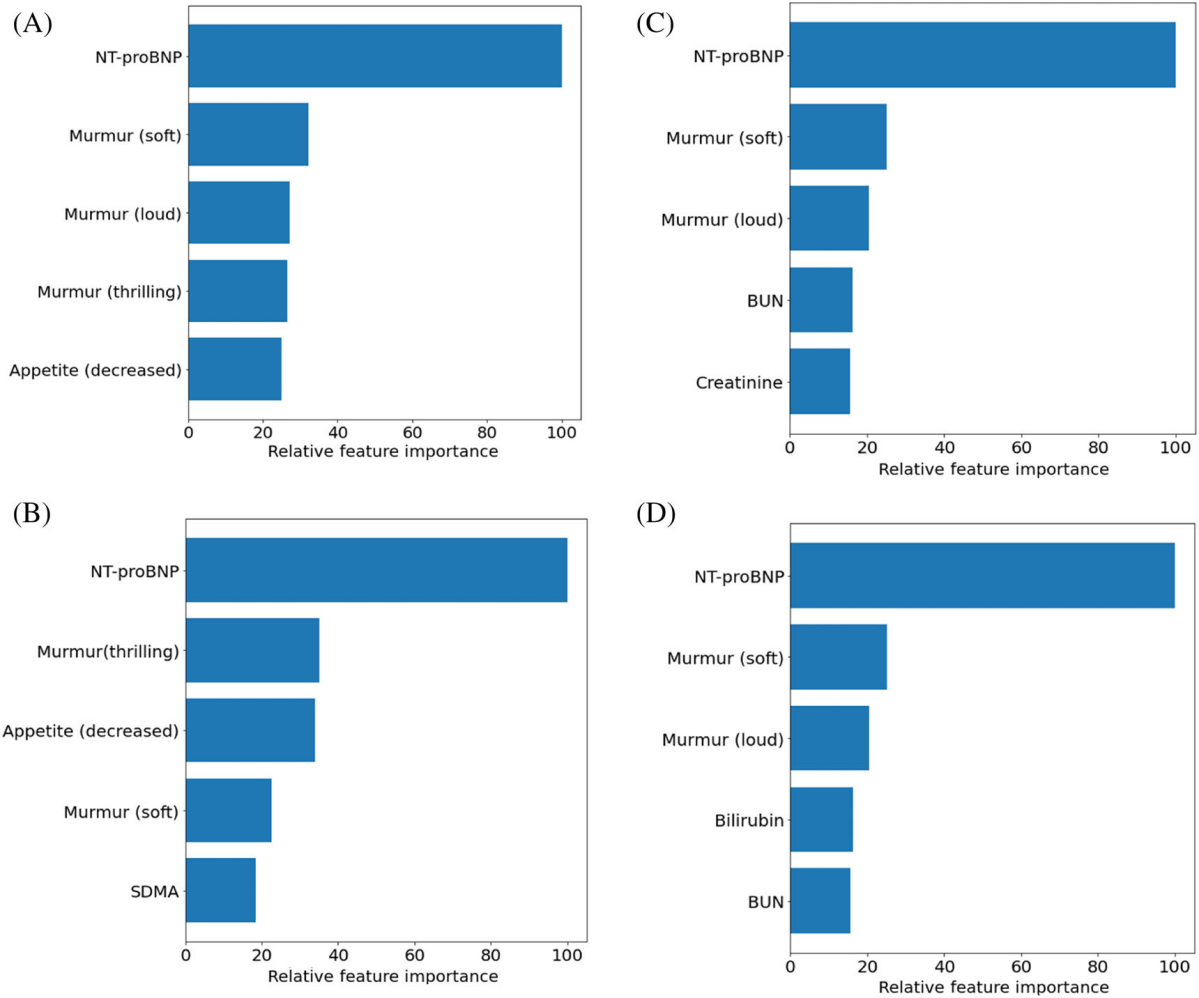
Variable importance plots displaying the 5 most important variables for the following classifiers: A, ridge regression; B, support vector machine with a linear kernel; C, Random Forest; and D, Gradient Boosting Machine (XGBoost). Variable importance is presented relative to the most important predictor in each model. Scores for ridge regression and the support vector machine with a linear kernel are the coefficients for each variable. Scores for random forest and gradient boosting machine are the mean Shapely value for each variable. BCS, body condition score; BUN, blood urea nitrogen; NT‐proBNP, N‐terminal propeptide of B‐type natriuretic peptide

When evaluating the AUC for predictions on the test data, multivariable model performance was relatively consistent across the different classification algorithm types with a mean value of 0.87, indicating that all models generalized well to new data. When NT‐proBNP was assessed as a sole predictor, the overall accuracy of the model was reduced. Performance metrics are summarized in Table 5. Both NT‐proBNP and murmur intensity were consistently found among the most important predictors, with NT‐proBNP ranking first in all models tested (Figure 6). These variables were featured in the predictive logistic regression model alongside appetite, creatinine, and BCS (Table 6). This predictive logistic regression model, with 5 features selected through backward stepwise elimination, performed similarly to the more complex explanatory model (AUC test, 0.86; 95% CI, 0.81‐0.91), supporting the relevance of appetite, BCS, serum creatinine concentration, murmur intensity, and NT‐proBNP concentration in capturing variation associated with disease stage.

**TABLE 5:** The performance of a series of models predicting preclinical disease status

		Multivariable logistic regression		SVM			Random forest	GBM	Logistic regression
		Backward stepwise elimination	Ridge regression	Linear	Polynomial	RBF		XGBoost	NT‐proBNP
Train (n = 848)	AUC (95% CI)	0.83 (0.80‐0.86)	0.85 (0.82‐0.88)	0.84 (0.81‐0.87)	0.84 (0.81‐0.87)	0.87 (0.84‐0.90)	0.97 (0.96‐0.98)	0.97 (0.96‐0.98)	0.78 (0.75‐0.82)
	Accuracy	0.81	0.82	0.81	0.81	0.83	0.89	0.91	0.78
	Bootstrap AUC (SD)	0.83 (0.017)	0.85 (0.016)	0.84 (0.017)	0.84 (0.017)	0.87 (0.015)	0.97 (0.005)	0.97 (0.006)	0.79 (0.019)
Test (n = 212)	AUC (95% CI)	0.86 (0.81‐0.91)	0.88 (0.83‐0.93)	0.88 (0.84‐0.93)	0.88 (0.83‐0.93)	0.87 (0.82‐0.92)	0.85 (0.80‐0.91)	0.86 (0.82‐0.91)	0.77 (0.70‐0.84)
	Accuracy	0.79	0.82	0.81	0.81	0.83	0.81	0.80	0.77
	Brier score	0.133	0.125	0.125	0.126	0.127	0.136	0.133	0.158
	Calibration in the large	−0.034	0.135	0.268	0.244	0.097	0.098	0.088	−0.056
	Calibration slope	1.129	1.441	1.492	1.454	1.383	1.275	1.217	0.950
n (variables required)		5	29	29	29	29	29	29	1
Interpretable		Yes	Yes	No	No	No	No	No	Yes

*Notes*: The 5 variables selected by backward stepwise elimination in the predictive logistic regression model were: appetite, body condition score, creatinine, murmur intensity, and NT‐proBNP. Model calibration was assessed using a locally weighted smoothing line (LOWESS) fitted to predicted probabilities plotted against the actual probability (prevalence) for each value. Support vector machine models were developed using linear, polynomial and radial basis function kernels. AUC, area under the receiver operating characteristic curve; CI, confidence intervals; GBM, gradient boosting machine; NT‐proBNP, N‐terminal propeptide of B‐type natriuretic peptide; RBF, radial basis function; SVM, support vector machine; XGBoost, extreme gradient boosting trees.

**TABLE 6:** The results of a predictive logistic regression model fitted on the training data with features selected by backward stepwise elimination

Variable	β	Odds ratio (95% CIs)	*P*
Intercept	−10.00	4.53 e^−5^ (4.26 e^−6^ ‐ 4.33 e^−4^)	<.001
Appetite (decreased)	2.99	19.81 (3.73‐154.60)	.001
BCS (≤3)	‐	‐	‐
BCS (4)	−1.83	0.16 (0.05‐0.52)	.003
BCS (5)	−1.39	0.25 (0.08‐0.77)	.02
BCS (6)	−1.11	0.33 (0.10‐1.04)	.06
BCS (7)	−1.58	0.21 (0.05‐0.74)	.02
BCS (≥8)	−2.01	0.13 (0.03‐0.58)	.009
Creatinine	−0.02	0.98 (0.97‐0.99)	<.001
Murmur (soft)	‐	‐	‐
Murmur (moderate)	0.73	2.07 (1.09‐4.18)	.03
Murmur (loud)	1.59	4.89 (2.59‐9.85)	<.001
Murmur (thrilling)	1.93	6.89 (3.19‐15.61)	<.001
Log_10_(NT‐proBNP)	3.66	38.98 (19.38‐81.56)	<.001

*Notes*: Creatinine underwent analysis in Systéme International (SI) units (μmol/L), so the coefficient reported reflects the change associated with a 1‐unit increase measured on this scale. BCS, body condition score; CI, confidence intervals; e, multiply by 10^x^; log_10_, logarithmic transformation to the base 10; NT‐proBNP, N‐terminal propeptide of B‐type natriuretic peptide; *P*, statistical significance; β, regression coefficient. A hyphen (‐) indicates that this category was used as a reference group for comparisons. The following levels of categorical variables were chosen as reference groups to provide a suitable point of comparison: appetite, not reduced; BCS, ≤3; murmur intensity, soft. For continuous variables that have been log transformed, the OR represents the change in odds associated with a 10‐fold increase in a variable.

## 4 DISCUSSION

Our study found that clinical observations and cardiac biomarker concentrations can be used to differentiate between dogs in stages B1 and B2. Furthermore, analyzing multiple variables at once results in a greater number of cases being correctly classified. With novel data, multivariable predictive models estimate the likelihood of being stage B2 with good discrimination (test set mean AUC, 0.87) and calibration.

There is a need to diversify the range of diagnostic options available in DMVD to better accommodate different circumstances. The predictive models defined in the present study have the potential as an initial screening test, quantifying the risk of having stage B2 disease. High‐risk scores could help select dogs that would benefit from further investigation and low‐risk scores could identify dogs that are more likely to be stage B1. In this study, a predictive model derived using multivariable logistic regression had similar performance to other, more complex methods. As this model uses fewer variables, barriers to uptake such as cost would be reduced. The model was internally validated against a holdout set of 20% of the cohort and on the basis of this analysis; it is possible to infer that it will perform well if applied to new cases. An important next step is to assess the model's accuracy in the exact set of circumstances in which it is intended for use; primary‐care practice.^38^ If satisfactory, this model could be used to support clinical decision making in preclinical DMVD. As the potential impact of this model is influenced by user uptake and engagement, we propose that it is presented in the form of an app. This digital medium would allow vets to engage with the full model through a graphical user interface which could perform calculations and assist in interpretation of results.

All predictive models ranked NT‐proBNP as the most important variable when differentiating between stages. In the explanatory analysis, the likelihood of having stage B2 disease increased as NT‐proBNP concentration increased, supporting previous associations with disease severity.^11^, ^12^, ^39^, ^40^ When comparing the multivariable explanatory model with a model containing NT‐proBNP alone, including other risk factors alongside the biomarker improved discriminatory performance, reducing the number of misclassified cases. Similar findings were also apparent in the results of predictive models (Supplementary Table 4). As well as capturing additional sources of variation in the outcome measure, a multivariable approach could have improved performance by controlling for variability in the biomarker itself. In dogs, NT‐proBNP concentrations are affected by comorbidities, display biological variability within individuals, and might be affected by breed.^41^, ^42^, ^43^, ^44^ Including more than 1 marker of disease severity could therefore improve the quality of predictions in cases with anomalous biomarker concentrations. The findings of our explanatory analysis are similar to previous studies, indicating that NT‐proBNP is more informative when interpreted alongside other factors.^5^, ^16^, ^17^ This results in improved accuracy when staging preclinical disease.

In addition to NT‐proBNP, several other risk factors were identified. Murmur intensity, another important predictive variable, is associated with preclinical disease severity.^20^, ^45^ Our study found that the likelihood of being in stage B2 increased with murmur grade, with dogs having loud or thrilling murmurs at the greatest risk. Murmur intensity is one of the more subjective measurements when compared to the other variables included in this analysis. Cardiac auscultation is subject to inter‐ and intraobserver variability, which is potentially limiting considering the apparent importance of this variable.^45^, ^46^ As the use of simpler schemes improves agreement, audibility was graded using a 4 level system that has been previously used to assess DMVD severity.^20^ This had the advantage of regrouping grades that did not occur very commonly, reducing the dimensionality of our data for analysis. The predictive models we describe use this system and demonstrate good predictive accuracy. It is still however important to note that all dogs were examined by veterinary cardiologists using a standardized protocol and further research is required to assess whether sampling in a different setting could impact the accuracy of results. The decision to use this system was informed by our research question and data, and we do not necessarily suggest that this replaces methods currently used in practice.

Having a reduced appetite was found to increase the likelihood of being in stage B2. In DMVD, loss of appetite is considered a negative prognostic indicator and some dogs that go on to develop CHF experience reductions in body weight.^4^, ^6^, ^47^ Although weight was not examined in the present analyses, poor BCS was associated with increased risk. As in humans,^48^, ^49^ cachexia might develop before the onset of CHF, resulting in changes that can be detected as clinical signs. In this study, a negative association was observed between creatinine and the odds of being stage B2, and creatinine was selected by several multivariable methods in preference to symmetric dimethylarginine (SDMA). As both variables vary with GFR, the selection of creatinine suggests that its association with disease severity could in part relate to cachexic losses in muscle mass. Glomerular filtration rate itself might be expected to display an association with the severity of preclinical disease as increases in circulating fluid volume have been shown to induce a more rapid rate of creatinine clearance.^50^ Adjusting for creatinine in a model containing NT‐proBNP is potentially advantageous as GFR is a confounder of the biomarker's concentrations.^51^

Although age and ALT were associated with the likelihood of being stage B2, neither variable was retained in the predictive model derived from a smaller subset of data. In explanatory analyses, the greatest risk was observed when dogs were between 8 and 10, and after this aging dogs were less likely be in stage B2. In humans, age is considered when defining diagnostic thresholds for NT‐proBNP and research has shown that this explains additional variation in analyses that already account for creatinine.^52^ There is evidence that the propensity to remodel is altered in the aging heart; however, this has not been studied in DMVD.^53^ It is possible that profibrotic changes in myocardial composition could affect the tendency of the heart to dilate.^54^ Alternatively, these findings could reflect differences in the phenotypes contained within each age group. Early onset DMVD, as noted in some breeds, might be accompanied by a more rapid rate of disease progression.^2^, ^5^ The explanatory analysis also identified ALT as a risk factor. As the hepatic vasculature is sensitive to changes in central venous pressure, elevations in ALT can occur in cardiovascular disease as a result of congestion or reduced perfusion.^55^ Alanine aminotransferase interacted with NT‐proBNP, although the exact relevance of this finding in DMVD is unclear. At high ALT concentrations, NT‐proBNP could be partially elevated as a consequence of liver disease, producing a weaker association with DMVD severity.^56^ In addition, 15 dogs were receiving treatment with corticosteroids which can affect both variables.^57^ This is not the first study to describe changes in ALT associated with the severity of preclinical disease;^6^ however, it is possible that this result was in some way confounded. Further research is needed to understand the mechanisms responsible for this finding.

### 4.1 Strengths and limitations

The study benefited from the large number of cases, which facilitated robust analyses, particularly when developing a predictive model for clinical use. When training any model, it is possible that the algorithm will overfit nonmeaningful noise in the data, reducing its generalisability.^58^ In this study, there were enough cases to simulate performance in novel conditions. Several algorithms were compared and there was good agreement in internal validity and the variables of greatest importance. A possible criticism, however, is that the conditions of examination are not those found in primary‐care practice. Data were collected from a referral sample by specialists following a protocol, and blood samples were analyzed at research laboratories. A follow‐on external validation study is required to assess whether predictions are reliable when these conditions are changed.

The data were substantial enough to evaluate several ML algorithms and present them in comparison with conventional regression models. Machine learning has potential applications in medicine as algorithms can describe complex, nonlinear relationships among variables.^59^ In this study, using ML to distinguish between stages did not produce a marked advantage in performance, so the model derived using logistic regression could be considered the most clinically useful as it is both parsimonious and interpretable. Although ML shows promise in veterinary medicine, these results highlight that it does not always provide an optimal solution; model selection is equally dependent upon the data and the model's intended use. It is however, worth noting that stepwise regression is not without limitations. Selection methods can be unstable and small increases in additivity might have been lost in favor of parsimony.^60^ In our study, this method was used to objectively reduce the number of variables required for prediction, rather than for causal inference. The output this model produces is the predicted probability that a dog is in stage B2. Decision‐making surrounding case management is likely to differ for dogs depending upon their score and this continuous outcome could help to answer a range of clinical questions. As previously discussed, predicted probabilities could be generated through the use of an app and presented alongside PPV, NPV, sensitivity, and specificity at different thresholds to aid interpretation (Table 7).

**TABLE 7:** Classification performance of a predictive multivariable logistic regression model at different thresholds of predicted probability

Predicted probability (%)	PPV (%)	NPV (%)	Sensitivity (%)	Specificity (%)
10	38.90 (37.24, 40.64)	95.51 (92.91, 97.72)	95.00 (92.08, 97.50)	41.12 (37.13, 45.07)
20	47.50 (44.62, 50.63)	89.93 (87.42, 92.34)	81.67 (77.67, 86.25)	64.31 (60.69, 67.93)
30	55.21 (51.23, 59.41)	86.90 (84.63, 89.28)	70.42 (64.58, 70.42)	77.47 (74.18, 80.60)
40	62.29 (57.20, 67.53)	84.57 (82.46, 86.69)	60.42 (54.17, 66.68)	85.53 (82.57, 88.32)
50	71.59 (65.88, 77.38)	83.51 (81.57, 85.45)	54.17 (47.50, 60.42)	91.45 (89.14, 93.59)
60	78.29 (71.65, 84.56)	80.89 (79.18, 82.65)	42.92 (36.37, 49.58)	95.25 (93.59, 96.88)
70	86.25 (79.00, 92.96)	78.20 (76.82, 79.71)	30.83 (25.00, 37.08)	98.03 (96.88, 99.01)
80	86.11 (75.00, 95.12)	74.69 (73.71, 75.72)	15.00 (10.42, 19.58)	99.01 (98.19, 99.67)
90	100 (100, 100)	72.73 (72.21, 73.34)	5.00 (2.50, 7.92)	100 (100, 100)

*Notes*: Incremental increases in the predicted probability were evaluated as the threshold used to classify dogs as being in stage B2. The utility of each threshold was assessed using training set data. Confidence intervals were calculated using 2000 stratified bootstrap replicates (R package “pROC” v1.16.2). CI, confidence interval; NPV, negative predictive value; PPV, positive predictive value.

Prospectively sampling a large number of dogs captured data from other diagnostic tests, allowing us to conduct a subanalysis of VHS; an alternative method of identifying cardiomegaly. As measurements of VHS were not required for inclusion in this study only a small number of dogs had VHS recorded, which increased the size of CIs and might have introduced bias if dogs that underwent radiography had more advanced DMVD or other signs that warranted thoracic imaging. In comparison with single tests like VHS or NT‐proBNP, this study found that a multivariable approach was more accurate, which concurs with the results of other studies,^5^, ^16^, ^17^, ^47^ and would reduce the number of misclassified cases. Integrating routine data with a blood test also avoids risk associated with radiation exposure or chemical restraint.^61^ Multiparameter approaches could offer a superior way to interpret VHS in instances where radiography is part of the diagnostic workup for preclinical disease.

Dogs receiving treatment with pimobendan were excluded from the clean data and, in doing this dogs with more severe disease could have been removed (Supplementary Tables 5 and 6).^5^ Dogs receiving drugs that inhibit the renin angiotensin aldosterone system (RAAS) were not excluded as to date, no study has conclusively demonstrated an effect of RAAS blockade on left‐sided cardiac dimensions in dogs. Applying the explanatory model to dogs that had not been included in primary analyses indicated that the performance of the model was reduced. The prediction model defined in this study should therefore be interpreted more cautiously if dogs are on pimobendan, have significant comorbidities, or if there are issues with sample handling. Other factors that could have affected the accuracy of our results include the use of complete case analysis, variation associated with individual practices, and the inclusion of dogs in CHF if this was not detected at the point of examination. It should also be noted that the associations described in our explanatory analysis represent findings present at a sample level. The large number of dogs examined increased our ability to detect these associations and, for some variables, similar findings would be challenging to appreciate within individuals. Finally, it is important to recognize that the predictive model defined in this study is diagnostic rather than prognostic. Although higher values of predicted probability could reflect more advanced preclinical disease, an association with outcome has not been assessed. In addition, the output of this model should not be considered a surrogate for appropriate echocardiographic testing if this is attainable.

## 5 CONCLUSION

This study found that variables from different aspects of a dog's examination could be used in combination to assess the likelihood of being stage B2. A predictive model that analyses a dog's appetite, BCS, creatinine concentration, murmur intensity, and NT‐proBNP concentration could be presented as an app for use in primary‐care practice. This has potential as a screening test and might provide an informed way to allocate client and practice resources. The correct application of this prediction model could improve outcomes for dogs with preclinical DMVD.

## CONFLICT OF INTEREST DECLARATION

The study was supported by Boehringer Ingelheim Animal Health GmbH who provided funding for the costs of the study directly and through sponsorship of the primary author's (J. Wilshaw) postgraduate studies. For individual dogs, the costs associated with analysis of blood samples were covered by the study. G. Wess has received funding from IDEXX for research and speaking. S.G. Gordon holds a consultancy with Boehringer Ingelheim Ltd has received research funding. She has received funding for travel, research, speaking and production of educational materials from IDEXX. J. Elliott is a paid consultant with Boehringer Ingelheim Ltd, CEVA Animal Health, and Elanco Animal Health. He has received grant funding from CEVA Animal Health and Elanco Animal Health. A. Boswood holds a consultancy with Boehringer Ingelheim Ltd and CEVA Animal Health. He has received research funding from Boehringer Ingelheim, CEVA Animal Health and Zoetis. Except for M.A. Oyama, J. Elliott and D. Xia, authors attended a writing meeting to discuss this manuscript and had travel costs covered by Boehringer Ingelheim. S. Rosenthal, D. Dickson, L. Bevilacqua, E. Dutton, M. Deinert, R. Abrantes, I. Schneider, M.A. Oyama, and D. Xia otherwise have no conflicts of interest to declare. A patent application has been filed by the Royal Veterinary College to protect the intellectual property associated with a predictive algorithm to identify dogs with stage B2 disease among preclinical cases.

## OFF‐LABEL ANTIMICROBIAL DECLARATION

Authors declare no off‐label use of antimicrobials.

## INSTITUTIONAL ANIMAL CARE AND USE COMMITTEE (IACUC) OR OTHER APPROVAL DECLARATION

The collection and storage of dog data were performed with owner consent, in accordance with relevant guidelines and regulations and with the approval of the Ethics and Welfare Committee of the Royal Veterinary College (URN: 2017 1749‐3).

## HUMAN ETHICS APPROVAL DECLARATION

Authors declare human ethics approval was not needed for this study.

## Supporting information


**Appendix S1**: Supporting information

**Appendix S2**: Supporting information
