# Supplementary material for: Accuracy of history, physical examination, cardiac biomarkers, and biochemical variables in identifying dogs with stage B2 degenerative mitral valve disease
Source: J Vet Intern Med. 2021 Mar 1;35(2):755–70. doi: 10.1111/jvim.16083 (PMC7995403; doi:10.1111/jvim.16083)
Supplement: Supplementary file 1 — Appendix S1: Supporting information [file JVIM-35-755-s001.pdf]

Supplementary Materials

Accuracy of history, physical examination, cardiac biomarkers and biochemical parameters in identifying stage B2 degenerative mitral valve disease

J. Wilshaw, S.L. Rosenthal, G. Wess, D. Dickson, L. Bevilacqua, E. Dutton, M. Deinert, R. Abrantes, I. Schneider, M.A. Oyama, S.G. Gordon, J. Elliott, D. Xia, and A. Boswood

Supplementary Methods: Data Pre-processing for Prediction.

The binary logistic regression was developed in R manually and using the package “rms” (v5.1-4). All machine learning models were developed using the Python library Scikit-learn (v0.23.1, packages: “linear-model”, “svm”, “ensemble”, “xgboost” v1.2.0). Transformation functions for data pre-processing were developed on the training set and applied to test data at the point of prediction. For all models except logistic regression, continuous variables were scaled using the formula $(x_i - \bar{x})/\ \sigma(x)$. This is a standard technique to ensure that variables are presented on the same scale; the number of standard deviations away from the mean, as different orders of magnitude can falsely influence model development. As SVM and decision tree algorithms do not assume linearity, variables were not quantile transformed for these models. Categorical variables were dummy encoded (*number of categories (k) – 1*) for logistic regression to label a category as the reference group, and one hot encoded for other algorithm types (k) to allow assessment of the importance of each level. The tuning metric for hyperparameter selection was the Brier score, which represents the mean squared error between predicted probabilities and the outcome.

Supplementary Table 1. Characteristics of dogs in the Clean, Complete and Confounded samples.

Variable		Sample		
		Clean (n = 1245)	Complete (n = 1887)	Confounded (n = 642)
Age (years)		10.00 (8.03, 11.63)	10.00 (8.17, 11.83)	10.33 (8.27, 12.17)
BCS	≤ 3	2.33% (29)	2.65% (50)	3.27% (21)
	4	15.98% (199)	16.59% (313)	17.76% (114)
	5	42.09% (524)	421.92% (791)	41.59% (267)
	6	24.02% (299)	23.04% (434)	21.03% (135)
	7	11.16% (139)	11.13% (210)	11.06% (71)
	≥ 8	3.93% (49)	6.6% (79)	4.67% (30)
Breed	CKCS	23.45% (292)	25.33% (478)	28.97% (186)
Sex	Female entire	3.69% (46)	3.71% (70)	3.74% (24)
	Female neutered	38.63% (481)	39.48% (745)	41.12% (264)
	Male entire	11.89% (148)	12.13% (229)	12.62% (81)
	Male neutered	45.78% (570)	44.67% (843)	42.52% (273)
Weight (kg)		9.10 (6.60, 12.30)	9.05 (6.50, 12.18)	9.00 (6.30, 11.90)

Legend: Descriptive statistics are reported as the median (LQ, UQ) for continuous variables and the proportion (frequency) for categorical variables. N represents the number of dogs belonging to a group. BCS, body condition score; CKCS, Cavalier King Charles Spaniel.

Supplementary Table 2. Clinicopathological data for dogs included in the Clean, Complete and Confounded samples.

Variable		Sample		
		Clean (n = 1245)	Complete (n = 1887)	Confounded (n = 642)
Appetite	Decreased	1.20% (15)	1.91% (36)	3.27% (21)
Cardiac biomarkers	cTnI (ng/mL)	0.05 (0.03, 0.09)	0.05 (0.03, 0.09)	0.05 (0.03, 0.10)
	NT-proBNP (pmol/L)	687.00 (426.00, 1121.00)	704.00 (427.00, 1171.00)	731.50 (434.75, 1261.00)
Cardiac medications	ACEi	3.45% (43)	10.28% (194)	23.52% (151)
	Pimobendan	-	19.13% (361)	56.23% (361)
	Spironolactone	0.72% (9)	2.60% (49)	6.23% (40)
Cough	Yes	24.02% (299)	25.86% (488)	29.44% (189)
Disease stage	B2	27.07% (337)	32.22% (608)	42.21% (271)
Exercise tolerance	Decreased	11.16% (139)	11.39% (215)	11.84% (76)
Heart rate		120.00 (108.00, 136.00)	120.00 (108.00, 136.00)	120.00 (108.00, 136.00)
Heart rhythm	Regular rhythm	64.50% (803)	64.86% (1224)	65.58% (421)
	Sinus arrhythmia	33.25% (414)	33.12% (625)	32.87% (211)
	Other	2.17% (27)	1.96% (37)	1.56% (10)
LA:Ao		1.48 (1.31, 1.71)	1.52 (1.33, 1.75)	1.60 (1.39, 1.83)
LVIDDN (cm/kg$^{0.294}$)		1.67 (1.52, 1.84)	1.71 (1.54, 1.89)	1.78 (1.60, 1.96)
Murmur intensity	Soft	19.84% (247)	17.22% (325)	12.15% (78)
	Moderate	39.20% (488)	36.25% (684)	30.53% (196)
	Loud	31.33% (390)	34.82% (657)	41.59% (267)
	Thrilling	9.40% (117)	11.55% (218)	15.73% (101)
Respiratory rate		26.00 (20.00, 32.00)	25.00 (20.00, 32.00)	24.00 (22.00, 32.00)
Serum biochemistry	Albumin (g/L)	33.00 (31.00, 35.00)	33.00 (31.00, 35.00)	33.00 (31.00, 35.00)
	ALKP (U/L)	55.00 (30.00, 139.00)	57.00 (30.00, 146.75)	61.00 (31.00, 176.00)
	ALT (U/L)	49.00 (35.00, 76.00)	51.00 (35.00, 78.00)	55.00 (36.00, 87.00)
	Bilirubin (µmol/L)	3.20 (2.20, 3.42)	3.20 (2.40, 3.42)	3.20 (2.40, 3.42)
	BUN (mmol/L)	6.07 (4.88, 7.70)	6.10 (4.90, 7.90)	6.41 (5.00, 8.57)
	Calcium (mmol/L)	2.50 (2.40, 2.60)	2.50 (2.40, 2.60)	2.50 (2.40, 2.60)
	Chloride (mmol/L)	111.00 (109.00, 113.00)	111.00 (109.00, 113.00)	111.00 (109.00, 113.00)

	Cholesterol (mmol/L)	6.19 (5.10, 7.40)	6.20 (5.10, 7.50)	6.30 (5.20, 7.70)
	Creatinine (μmol/L)	64.00 (53.04, 79.00)	64.00 (53.04, 79.56)	65.00 (53.04, 79.56)
	GGT (U/L)	4.00 (3.00, 6.00)	4.00 (3.00, 6.00)	4.00 (3.00, 6.00)
	Globulin (g/L)	30.00 (28.00, 33.00)	30.00 (28.00, 33.00)	30.00 (28.00, 33.00)
	Glucose (mmol/L)	5.30 (4.80, 5.70)	5.30 (4.80, 5.71)	5.26 (4.70, 5.77)
	Phosphate (mmol/L)	1.20 (1.00, 1.40)	1.20 (1.03, 1.40)	1.29 (1.10, 1.50)
	Potassium (mmol/L)	4.50 (4.30, 4.80)	4.50 (4.30, 4.80)	4.60 (4.30, 4.80)
	SDMA (μg/dL)	10.00 (9.00, 12.00)	10.00 (9.00, 13.00)	11.00 (9.00, 13.00)
	Sodium (mmol/L)	148.00 (147.00, 150.00)	148.00 (147.00, 150.00)	148.00 (147.00, 150.00)
VHS		n = 175, 11.00 (10.50, 11.50)	n = 267, 11.00 (10.50, 11.80)	n = 92, 11.15 (10.50, 12.00)

Legend: Descriptive statistics are reported as the median (LQ, UQ) for continuous variables and the proportion (frequency) for categorical variables. N represents the number of dogs belonging to a group. ACEi, angiotensin converting enzyme inhibitor; cTnI, cardiac troponin I; NT-proBNP, N-terminal propeptide of B-type natriuretic peptide; ALKP, alkaline phosphatase; ALT, alanine aminotransferase; BUN, blood urea nitrogen; GGT, gamma-glutamyl transferase; SDMA, symmetric dimethylarginine; VHS, vertebral heart score.

Supplementary Table 3. Results of univariable logistic regression models for the explanatory analysis of risk factors associated with stage B2 DMVD.

Variable	*P* Value
Age	0.018
Albumin	0.973
Log_{10}(ALKP)	0.013
Log_{10}(ALT)	0.186
Appetite	0.007
BCS	0.001
Bilirubin	0.111
Breed	0.298
BUN	0.387
Calcium	0.251
Chloride	0.935
Cholesterol	0.006
Cough	< 0.001
Creatinine	0.005
cTnI	< 0.001
Exercise tolerance	0.005
Log_{10}(GGT)	0.223
Globulin	0.390
Glucose	0.811
Heart rate	< 0.001
Heart rhythm	0.003
Laboratory	0.496
Murmur intensity	< 0.001
Log_{10}(NT-proBNP)	< 0.001
Phosphate	0.004
Potassium	< 0.001
Respiratory rate	0.103
SDMA	0.230
Sex	0.464
Sodium	0.657

Legend: Variables where $P < 0.2$ were selected for multivariable analysis. Age, bilirubin and cTnI had been quartile transformed and tested as categorical variables due to violation of the assumption of linearity. cTnI, cardiac troponin I; NT-proBNP, N-terminal propeptide of B-type natriuretic peptide; ALKP, alkaline phosphatase; ALT, alanine aminotransferase; BUN, blood urea nitrogen; GGT, gamma-glutamyl transferase; SDMA, symmetric dimethylarginine.

Supplementary Table 4: Classification performance at different thresholds of predicted probability. Performance for the predictive logistic model is presented in comparison to a model containing NT-proBNP alone.

Predicted Probability (%)	Model	PPV (%)	NPV (%)	Sensitivity (%)	Specificity (%)
10	Multivariable	38.90 (37.24, 40.64)	95.51 (92.91, 97.72)	95.00 (92.08, 97.50)	41.12 (37.13, 45.07)
	NT-proBNP (391 pmol/L)	33.73 (32.50, 35.05)	91.86 (87.75, 95.45)	93.75 (90.42, 96.67)	27.30 (23.68, 30.92)
20	Multivariable	47.50 (44.62, 50.63)	89.93 (87.42, 92.34)	81.67 (77.67, 86.25)	64.31 (60.69, 67.93)
	NT-proBNP (635 pmol/L)	41.95 (39.34, 44.67)	88.86 (85.86, 91.58)	82.50 (77.50, 87.08)	54.93 (50.82, 59.05)
30	Multivariable	55.21 (51.23, 59.41)	86.90 (84.63, 89.28)	70.42 (64.58, 70.42)	77.47 (74.18, 80.60)
	NT-proBNP (877 pmol/L)	53.17 (49.07, 57.48)	85.97 (83.60, 88.27)	68.33 (62.50, 74.17)	76.32 (72.86, 79.61)
40	Multivariable	62.29 (57.20, 67.53)	84.57 (82.46, 86.69)	60.42 (54.17, 66.68)	85.53 (82.57, 88.32)
	NT-proBNP (1143 pmol/L)	60.82 (54.84, 66.67)	81.92 (80.00, 83.98)	51.25 (45.00, 57.92)	86.84 (83.88, 89.48)
50	Multivariable	71.59 (65.88, 77.38)	83.51 (81.57, 85.45)	54.17 (47.50, 60.42)	91.45 (89.14, 93.59)
	NT-proBNP (1457 pmol/L)	68.29 (61.07, 75.69)	79.46 (77.81, 81.24)	39.17 (32.92, 45.83)	92.76 (90.62, 94.74)
60	Multivariable	78.29 (71.65, 84.56)	80.89 (79.18, 82.65)	42.92 (36.37, 49.58)	95.25 (93.59, 96.88)
	NT-proBNP (1857 pmol/L)	73.75 (64.63, 82.76)	76.85 (75.54, 78.32)	26.67 (21.25, 32.50)	96.22 (94.73, 97.70)
70	Multivariable	86.25 (79.00, 92.96)	78.20 (76.82, 79.71)	30.83 (25.00, 37.08)	98.03 (96.88, 99.01)
	NT-proBNP (2419 pmol/L)	78.69 (66.67, 88.68)	74.87 (73.85, 76.08)	16.67 (12.08, 21.67)	98.19 (97.04, 99.18)
80	Multivariable	86.11 (75.00, 95.12)	74.69 (73.71, 75.72)	15.00 (10.42, 19.58)	99.01 (98.19, 99.67)
	NT-proBNP (3339 pmol/L)	77.27 (56.25, 94.12)	72.91 (72.27, 73.63)	6.67 (3.75, 10.00)	99.18 (98.36, 99.84)
90	Multivariable	100 (100, 100)	72.73 (72.21, 73.34)	5.00 (2.50, 7.92)	100 (100, 100)
	NT-proBNP (5426 pmol/L)	100 (100, 100)	72.04 (71.78, 72.38)	1.67 (0.42, 3.33)	100 (100, 100)

Legend: Incremental increases in the predicted probability were evaluated as the threshold used to classify dogs as being in stage B2. The utility of each threshold was assessed using training set data. Confidence intervals were calculated using 2000 stratified bootstrap replicates (R package "pROC" v1.16.2). Untransformed values of NT-proBNP for each predicted probability were calculated using the intercept and slope of a regression equation that had been fitted to the training set, and results were rounded to the nearest whole number. CI, confidence interval; PPV, positive predictive value; NPV, negative predictive value.

Supplementary Table 5. Characteristics of stage B2 dogs that were and were not receiving treatment with pimobendan.

Variable		No Pimobendan (n = 337)	Pimobendan (n = 204)
Age (years)		10.00 (8.50, 11.35)	10.00 (8.17, 11.83)
BCS	≤ 3	3.86% (13)	3.92% (8)
	4	14.24% (48)	22.06% (45)
	5	43.32% (146)	40.20% (82)
	6	28.49% (96)	17.16% (35)
	7	6.82% (23)	11.27% (23)
	≥ 8	2.67% (9)	4.90% (10)
Breed	CKCS	25.52% (86)	26.96% (55)
Sex	Female entire	2.67% (9)	2.94% (6)
	Female neutered	37.39% (126)	41.67% (85)
	Male entire	11.28% (38)	8.82% (18)
	Male neutered	48.66% (164)	46.57% (95)
Weight (kg)		8.70 (6.50, 11.30)	7.93 (5.90, 10.76)

Legend: Stage B2 dogs that were receiving treatment with pimobendan were selected from the "Confounded" sample. Data are presented alongside those from stage B2 dogs in the clean sample that were not receiving treatment with pimobendan. Descriptive statistics are reported for as the median (LQ, UQ) for continuous variables and the proportion (frequency) for categorical variables. N represents the number of dogs belonging to a group. BCS, body condition score; CKCS, Cavalier King Charles Spaniel.

Supplementary Table 6. Clinicopathological data for stage B2 dogs that were and were not receiving treatment with pimobendan

Variable		No Pimobendan (n = 337)	Pimobendan (n = 204)
Appetite	Decreased	2.67% (9)	2.45% (5)
Cardiac biomarkers	cTnI (ng/mL)	0.06 (0.04, 0.10)	0.06 (0.04, 0.10)
	NT-proBNP (pmol/L)	1188.00 (774.00, 2000.00)	1030.00 (650.25, 1746.50)
Cardiac medications	ACEi	3.56% (12)	43.14% (88)
	Spironolactone	1.48% (5)	16.67% (34)
Cough	Yes	34.4% (116)	41.18% (84)
Exercise tolerance	Decreased	15.43% (52)	10.78% (22)
Heart rate		128.00 (117.00, 140.00)	128.00 (111.50, 140.00)
Heart rhythm	Regular rhythm	71.81% (242)	75.49% (154)
	Sinus arrhythmia	26.41% (89)	22.06% (45)
	Other	1.48% (5)	2.45% (5)
LA:Ao		1.85 (1.72, 2.02)	1.84 (1.70, 2.00)
LVIDDN (cm/kg$^{0.294}$)		1.93 (1.81, 2.11)	1.95 (1.85, 2.09)
Murmur intensity	Soft	4.45% (15)	0.10% (2)
	Moderate	25.82% (87)	18.14% (37)
	Loud	48.66% (164)	53.92% (110)
	Thrilling	21.07% (71)	26.96% (55)
Respiratory rate		26.00 (22.00, 32.00)	28.00 (24.00, 30.00)
Serum biochemistry	Albumin (g/L)	33.00 (30.00, 35.00)	33.00 (31.00, 35.00)
	ALKP (U/L)	61.00 (33.00, 173.00)	66.00 (32.50, 195.00)
	ALT (U/L)	51.00 (37.00, 76.00)	59.00 (36.00, 91.50)
	Bilirubin (μmol/L)	3.10 (2.10, 3.42)	3.20 (2.30, 3.42)
	BUN (mmol/L)	5.90 (5.00, 7.50)	7.00 (5.30, 8.57)
	Calcium (mmol/L)	2.50 (2.40, 2.60)	2.45 (2.36, 2.60)
	Chloride (mmol/L)	111.00 (109.00, 113.00)	111.00 (109.00, 113.00)
	Cholesterol (mmol/L)	5.90 (4.86, 7.12)	6.08 (5.06, 7.60)
	Creatinine (μmol/L)	61.88 (52.00, 73.00)	65.00 (53.04, 79.56)

	GGT (U/L)	4.00 (3.00, 6.00)	4.00 (3.00, 6.00)
	Globulin (g/L)	30.00 (27.00, 33.00)	29.00 (28.00, 32.00)
	Glucose (mmol/L)	5.30 (4.90, 5.66)	5.04 (4.59, 5.60)
	Phosphate (mmol/L)	1.26 (1.10, 1.42)	1.26 (1.10, 1.49)
	Potassium (mmol/L)	4.60 (4.30, 4.90)	4.60 (4.40, 4.90)
	SDMA (μg/dL)	10.00 (9.00, 12.00)	10.00 (9.00, 12.00)
	Sodium (mmol/L)	148.00 (147.00, 150.00)	148.00 (147.00, 150.00)
VHS		n = 78, 11.50 (11.00, 12.25)	n = 32, 11.90 (11.00, 12.35)

Legend: Stage B2 dogs that were receiving treatment with pimobendan were selected from the "Confounded" sample. Data are presented alongside those from stage B2 dogs in the clean sample that were not receiving treatment with pimobendan. Descriptive statistics are reported as the median (LQ, UQ) for continuous variables and the proportion (frequency) for categorical variables. N represents the number of dogs belonging to a group. ACEi, angiotensin converting enzyme inhibitor; cTnI, cardiac troponin I; NT-proBNP, N-terminal propeptide of B-type natriuretic peptide; ALKP, alkaline phosphatase; ALT, alanine aminotransferase; BUN, blood urea nitrogen; GGT, gamma-glutamyl transferase; SDMA, symmetric dimethylarginine; VHS, vertebral heart score.

Supplementary Figure 1. A box and whisker plot displaying the distribution of values of NT-proBNP in ACVIM stages B1 and B2 in the clean sample.

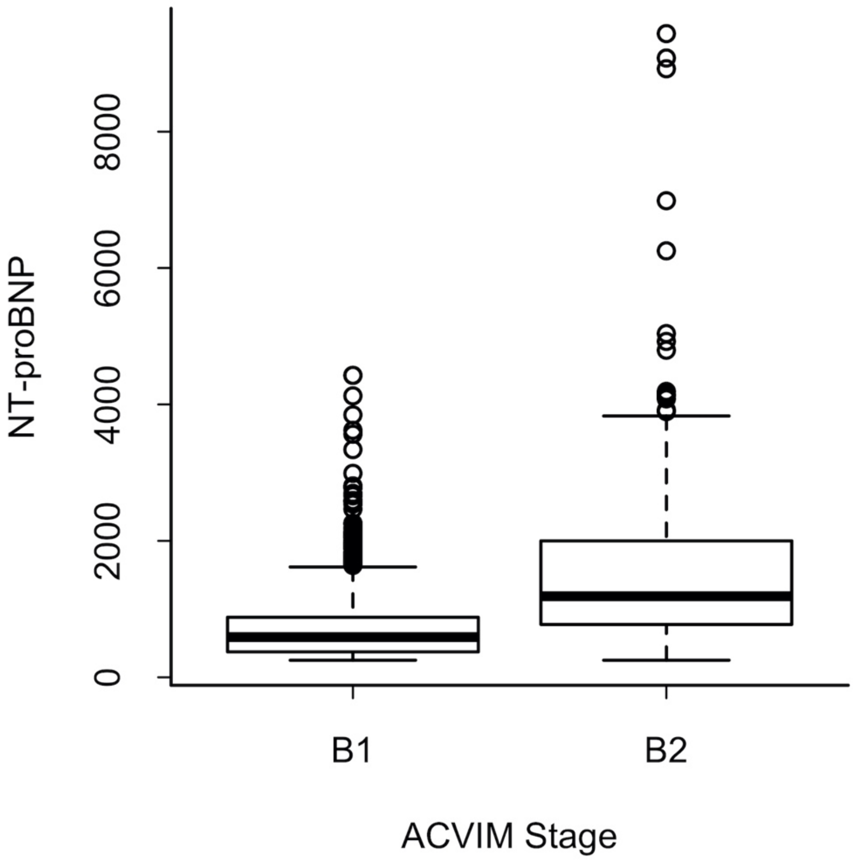

Legend: Disease stages are derived from the guidelines produced by the American College of Veterinary Internal Medicine's cardiology consensus panel. Dogs with LA:Ao ≥ 1.6 and LVIDDN ≥ 1.7 were classified as having stage B2 disease. NT-proBNP was measured in pmol/L. Boxes are used to display the interquartile range, divided by a line representing the median. Unfilled dots indicate values that fall outside of the median ± 1.5 x interquartile range.

Supplementary Figure 2. Slopes for the interaction between ALT and NT-proBNP.

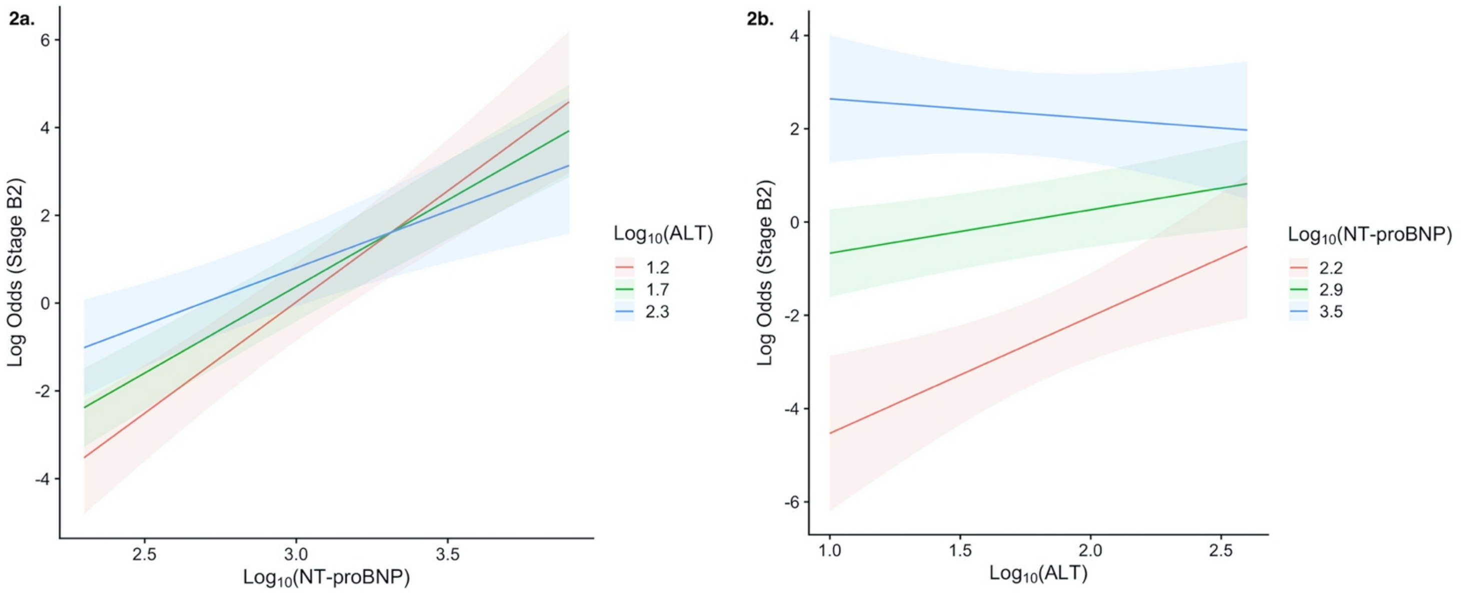

2a. NT-proBNP is plotted against the log odds of having stage B2 disease at different levels of ALT. *Legend:* The regression line for the interaction is red when log10(ALT) is 1.2 (mean – 2 x standard deviation), green when log10(ALT) is 1.7 (mean) and blue when log10(ALT) is 2.3 (mean + 2 x standard deviation). 95% confidence intervals are indicated by shading in the same colour. ALT, alanine aminotransferase; NT- proBNP, N-terminal propeptide of B-type natriuretic peptide; log10, logarithmic transformation to the base 10.

2b. ALT is plotted against the log odds of having stage B2 disease at different levels of NT-proBNP. *Legend:* The regression line for the interaction is red when log10(NT-proBNP) is 2.2 (mean – 2 x standard deviation), green when log10(NT-proBNP) is 2.9 (mean) and blue when log10(NT-proBNP) is 3.5 (mean + 2 x standard deviation). 95% confidence intervals are indicated by shading in the same colour. ALT, alanine aminotransferase; NT-proBNP, N-terminal propeptide of B-type natriuretic peptide; log10, logarithmic transformation to the base 10.
