# Supplementary material for: Accuracy of history, physical examination, cardiac biomarkers, and biochemical variables in identifying dogs with stage B2 degenerative mitral valve disease
Source: J Vet Intern Med. 2021 Mar 1;35(2):755–70. doi: 10.1111/jvim.16083 (PMC7995403; doi:10.1111/jvim.16083)
Supplement: Supplementary file 2 — Appendix S2: Supporting information [file JVIM-35-755-s002.pdf]

The HAMLET study is a prospective, multicentre, international, cross-sectional study which will obtain data from dogs with pre-clinical myxomatous mitral valve disease (MMVD).

STUDY OBJECTIVES

The objective of HAMLET is to determine how accurately a combination of clinical criteria and biomarker concentrations can discriminate dogs with stage B2 MMVD from those with stage B1 MMVD. The purpose of this is to develop an accessible diagnostic test to assist veterinary surgeons, at all skill levels, appropriately evaluate dogs with pre-clinical MMVD. By identifying dogs at high risk of having cardiac enlargement, practitioners could focus their efforts on encouraging the owners of those patients to pursue further evaluation e.g. echocardiography and if indicated, begin treatment with pimobendan.[1]

METHODS

Study Design

The study is cross-sectional in design. Patients with pre-clinical MMVD will be prospectively recruited by veterinary cardiologists at participating centres in Europe and the USA.

Data will be recorded and submitted by veterinary cardiologists with at least one of the following qualifications or trainees under direct supervision of suitably qualified cardiologists:

- Diplomate of the Cardiology Subspecialty of either the European College of Veterinary Internal Medicine (Companion Animal) or American College of Veterinary Internal Medicine
- RCVS Cardiology certificate or diploma (UK)
- Membership of the Collegium Cardiologie

The study population will comprise of dogs that are already visiting a veterinary cardiologist for diagnostic investigations to characterise and stage their MMVD.

Inclusion Criteria

Dogs are considered eligible for inclusion if they meet the following criteria:

1. They are 6 years of age or older
2. They weigh between ≥ 2 kg and ≤ 25 kg (≥4.4 and ≤ 55 lbs)
3. They have a left apical systolic murmur with maximum intensity over the mitral valve
4. Echocardiographic lesions characteristic of MMVD:
   - Valvular thickening and/ or prolapse of the mitral valve and associated apparatus
   - Evidence of mitral regurgitation on colour Doppler echocardiography
5. Accurate echocardiographic measurements of left atrial, aortic and left ventricular dimensions are available.

Exclusion criteria

Dogs are excluded from the study if they meet any of the following criteria:

1. They have clear radiographic, historical or physical examination signs consistent with congestive heart failure.
2. They are receiving diuretic medication.
3. They have a cardiac disease other than MMVD (allowing for concurrent tricuspid regurgitation).
4. Another significant systemic disease is already known to be present which may interfere with echocardiographic measurements obtained or be expected to affect biomarker concentrations.

This is a cross-sectional study and therefore data from any individual patient may only be entered once.

Study Duration

Projected study enrolment is 1000 subjects over a 12 month period.

Client Consent

The clinical information collected for the HAMLET study does not require additional investigation beyond the current recommendations outlined in the 2009 ACVIM consensus statement[2]. Informed and written consent for the procedures outlined below should therefore be gained from owners as part of the usual clinical proceedings. The patient forename and owner surname will be recorded on the laboratory request form to allow co-investigators to accurately identify and report these results to owners. It is the responsibility of the participating veterinarian to inform the client that the primary investigators will receive a copy of the laboratory results containing this information. This personal data will not be entered onto the study database. Owners that do not wish to have this information shared with the primary investigators will be allowed to opt out of participating in the study.

This protocol has received ethical approval from the Royal Veterinary College's Welfare and Ethics Committee & is in accordance with data protection law. A consent form for the study is provided in appendix 8 if co-investigators wish to use one, although completion is not compulsory.

Procedure

Co-investigators will be expected to evaluate and select subjects for enrolment using the inclusion and exclusion criteria listed above. All procedures listed below will be completed by the co-investigating cardiologist or a competent trainee (e.g. a resident) under the direct supervision of the investigator.

The use of sedative or anaesthetic agents does not preclude a patient from participating in the study. Their use is to be determined by the co-investigator in consultation with the owner and should reflect the best interests of the patient. If used, co-investigators should record this in the "Medication" section of the online data capture form.

Data collection is dependent upon completion of the following clinical procedures:

Case History

Co-investigators will obtain a full clinical history from clients on the same day as the diagnostic procedures are undertaken. Information about the patient's exercise tolerance (over the past 6 months), the presence of a cough (over the past 6 months), appetite (over the past 6 months) should be recorded on the standard data capture form along with noting other current co-morbidities and medications (both cardiac and non-cardiac) that the dog is currently receiving (see appendix 4).

Physical Examination

All patients will undergo a full physical examination. Heart rate, predominant heart rhythm and respiratory rate (expressed as beats per minute and breaths per minute respectively) will be measured during the consultation and recorded on the online data capture form. Murmur intensity should be graded using the Levine scale, attributing a value from I – VI (see appendix 1).[3] Patients will be weighed (weight and units of body weight will be recorded) and body condition will be scored using a nine-point scale provided in the protocol appendix (see appendix 2).

Echocardiography

Echocardiography will be performed on dogs positioned in right lateral recumbency.

The following measurements are required from routine right parasternal views:

- ***Short axis left atrial (LA) and aortic (Ao) diameter*** will be measured according to the method described by Hansson and others 2002:[4]

  The transverse diameters of the aorta and the left atrium are to be measured in a 2D, right parasternal short-axis view.

  For the aorta, the first calliper is placed at the midpoint of the convex curvature of the wall of the right aortic sinus. The calliper cross is positioned as close as possible to the blood-tissue interface. The second calliper is positioned at the point where the aortic wall and the non-coronary and left coronary aortic cusps merge. This measurement point is defined by a slight increase in echogenicity where the three structures merge. The left atrium is measured from this point by extending the aortic line to the blood-tissue interface of the LA wall. If a pulmonary vein enters the LA at the desired measurement point, the calliper is placed either on an extrapolation of the atrial border or immediately medial or lateral to the vein.

  The measurements are performed in early ventricular diastole using the first frame after aortic ejection where the aorta appears as a symmetric three-leaf clover with closed aortic valves and a teardrop shaped LA. In the same frame, the closed pulmonary valve leaflets may be seen to a variable extent. Measurements of LA and aortic root diameter should be taken from the same frame.

  Each measurement will be recorded from 3 cardiac cycles. The mean of 3 measurements of LA diameter and the mean of 3 measurements for the aortic root are to be recorded on the case report forms.

- ***Short axis, M-mode, left ventricular internal diameter in diastole (LVIDD) non-normalised***

  Left ventricular internal diastolic diameter measurements will be obtained from an M-mode recording of a right parasternal, short axis view of the left ventricle at the level of the chordae tendinae below the level of the mitral valve. Callipers should be placed on the leading edge of the endocardium of the inter-ventricular septum and the leading edge of the endocardium of the left ventricular free wall. Measurements will be reported in centimetres. As above, the mean of three measurements will be recorded on case report forms. Investigators are not required to index this measurement to bodyweight. For the purposes of analyses left ventricular diastolic diameter will be

normalised by the primary investigators according to the formula LVIDDN = LVIDD (cm)/$Wt^{0.294}$ (kg)

Vertebral Heart Size (If Available)

Patients are not required to complete this step to be included in the study. However if the co-investigator obtains a right lateral thoracic radiograph as part of their diagnostic investigation, the heart size should be assessed using the vertebral heart size (VHS) technique[5,6] and recorded on the online data capture form. To do this, co-investigators will measure the long axis of the cardiac silhouette using the heart base and apex as reference points. The short axis will be measured perpendicular to the long axis measurement where the cardiac silhouette is widest. Starting at the cranial edge of the fourth vertebra, the number of vertebral bodies, and their caudal disks, encompassed by the long axis measurement should be counted (measurement A). Similarly, the number of vertebral bodies, and their caudal disks, encompassed by the short axis measurement should be counted (measurement B). The VHS = measurement A + measurement B and should be reported to one decimal place.

Blood Sample Collection and Processing

A minimum of 5.0 ml of blood should be collected by venepuncture. Patients should ideally be fasted prior to sampling. If they have not been fasted please note this on the laboratory form and online data capture form.

IDEXX Laboratories will provide co-investigating centres with a sample collection pack. This pack contains all tubes, shipment materials and written instructions for sample processing. A copy of these instructions can be found in appendix 6.

Blood samples will be divided and placed into tubes as follows:

- 1.5ml serum, divided into one 1.0 ml aliquot and one 0.5 ml aliquot
- 0.3 ml EDTA plasma

Once samples are correctly placed into tubes, the provided barcode stickers should be used to label the samples. Label all tubes belonging to a single patient with stickers containing the same number. Put the labelled tubes in a transparent specimen bag, lined with an absorbent padding material. Place this into the provided container, along with a frozen cooling pad and the completed sample submission form.

To submit samples to IDEXX, co-investigators should arrange a DHL courier to collect the sample on the same day as the sample was obtained. This is provided at no extra expense to participants. If German sites already subscribe to an IDEXX courier, this method can be used for sample submission. All blood products should be stored at -20 °C until collected by a courier.

For further information regarding sample processing and courier collection, please refer to appendix 6 where an example set of instructions can be found. Following registration, these instructions will reflect each co-investigator’s location and available in-house laboratory equipment.

If co-investigators anticipate that they will run out of sample processing materials, they should contact the primary investigators via email at hamlet@rvc.ac.uk to request additional materials.

Blood Test Results

The results of amino terminal pro-B-type natriuretic peptide (NT-proBNP) concentrations and the biochemistry panel (analytes as outlined in appendix 5) will be reported back to investigators as they become available. Once results are finalised, a PDF report will be sent to the submitting centre and copied to the principal investigators.

The turnaround time of biochemistry and NT-proBNP panels is 24 hours following the receipt of samples. Co-investigators should expect to receive the results within 72 hours of sample submission.

Serum cardiac troponin I (cTnI) will also be measured as part of the study but all samples will be stored at -80°C for batch analysis at the end of the recruitment phase of the study. It is anticipated that recruitment for this study will be completed within 6 – 12 months. Co-investigators should not expect to receive cTnI results before this period of time has elapsed.

Residual Sample Storage

IDEXX Laboratories will store all residual samples at -20°C until the end of the recruitment phase of the study.

Ethical Approval

The study has received ethical approval from the Royal Veterinary College's Welfare and Ethics Committee. Centres should seek independent ethical approval from their own institution if they feel this is necessary. A consent form has been provided in appendix 8 if co-investigators wish to include this step when submitting for independent ethical approval.

DATA

Data Submission

Prior to enrolling participants, centres are asked to create an account on **www.rvc.ac.uk/hamlet.** The same account should be used by a centre each time they wish to enrol a patient in the study and therefore, a centre will only need to register once prior to recruitment of cases. Centres should not enrol any participants until they have registered for an account and have received project materials from IDEXX Laboratories.

To enrol a patient, co-investigators are required to complete an online data collection form (found at www.rvc.ac.uk/hamlet). On the form, investigators must record data from the patient's case history and physical examination, echocardiographic measurements of left

atrial, aortic and left ventricular size, and if available, vertebral heart size. The form can be partially completed in order to generate a patient case number before echocardiographic measurements are available but investigators will be prompted to complete forms in which all data has not been entered. After submission, co-investigators can view but not edit their completed forms. The site is hosted using a HTTPS server, meaning that all saved data is encrypted.

A copy of the online data collection form can be found in appendix 4. Investigators should read this prior to data collection.

If there are problems with the online data collection co-investigators should inform the primary investigators (hamlet@rvc.ac.uk) and manually complete a copy of the form (appendix 7) to send by email to hamlet@rvc.ac.uk or fax to 0207 468 5460.

Subject Identification

All enrolled subjects will have unique case number allocated to them at the time of completion of the online data collection form. Patients should subsequently be identified by this unique number on the laboratory sample submission form provided by IDEXX. The case number is provided to match patient data with the correct blood test results and maintain patient confidentiality in the study database.

Data Interpretation & Management

Data interpretation, management and analysis will be undertaken by the principal investigators at the Royal Veterinary College, London.

Grouping

Subjects will be classified by the principal investigators as either stage B1, stage B2, or equivocal stage B MMVD. An individual will be defined as having stage B2 if they meet both

of the following criteria. Dogs meeting only one of the criteria will be considered "equivocal" and analysed as a separate group in sub-analyses:

- LA:Ao ≥ 1.6
- LVIDDn ≥ 1.7

Statistical analysis

The primary outcome of the study will be the construction of a multivariable logistic regression model to predict the echocardiographic status of the dog based on a combination of clinical and biochemical biomarkers. The outcome will be dichotomous, each dog will be categorised according to whether or not they meet the previously defined stage B2 criteria.

Secondary outcomes will include more detailed evaluation of factors that influence NT-proBNP concentration in a population of dogs with MMVD. Assuming that the results meet the assumptions of the model, multivariable linear regression will be used to evaluate the relationship between NT-proBNP concentrations and multiple independent explanatory variables.

Use of Data

The results of this study are intended for publication in a peer-reviewed journal. Co-investigating cardiologists will be acknowledged for their participation.

Results may be presented by the primary investigators at veterinary conferences or used for teaching purposes.

COSTS COVERED BY THE STUDY

The blood tests listed in appendix 5 will be performed at no cost to clients or co-investigators. The costs of all other clinical procedures above (i.e. history taking, physical

examination and echocardiography) will not be covered by the study, as it is anticipated that enrolled dogs will undergoing these as part of the evaluation by the attending cardiologist.

CONFLICT OF INTEREST

The HAMLET study is funded by Boehringer Ingelheim Animal Health Gmbh.

References

1. Boswood A, Häggström J, Gordon SG, Wess G, Stepien RL, Oyama MA, et al. Effect of Pimobendan in Dogs with Preclinical Myxomatous Mitral Valve Disease and Cardiomegaly: The EPIC Study-A Randomized Clinical Trial. J Vet Intern Med. 2016 Nov;30(6):1765–79.
2. Atkins C, Bonagura J, Ettinger S, Fox P, Gordon S, Haggstrom J, et al. Guidelines for the diagnosis and treatment of canine chronic valvular heart disease. J Vet Intern Med. 2009;23(6):1142–50.
3. Levine SA. Notes on the Gradation of the Intensity of Cardiac Murmurs. JAMA. 1961 Jul 29;177(4):261.
4. Hansson K, Häggström J, Kvart C, Lord P. Left atrial to aortic root indices using two-dimensional and M-mode echocardiography in cavalier King Charles spaniels with and without left atrial enlargement. Vet Radiol Ultrasound. 43(6):568–75.
5. Buchanan JW, Bücheler J. Vertebral scale system to measure canine heart size in radiographs. J Am Vet Med Assoc. 1995 Jan 15;206(2):194–9.
6. Hansson K, Häggström J, Kvart C, Lord P. Interobserver variability of vertebral heart size measurements in dogs with normal and enlarged hearts. Vet Radiol Ultrasound. 2005;46(2):122–30.
7. Cave N, Zealand N, Canada CM, France PN, Uk RT. WSAVA nutritional assessment guidelines. Compend Contin Educ Vet. 2011;13(August):516–25.

APPENDICES

1. Levine Murmur Grading Scale [3]

Intensity	Description	Thrill present
Grade I	Faint, quieter than heart sounds	No
Grade II	Readily audible, quieter than heart sounds	No
Grade III	Moderate, as loud as heart sounds	No
Grade IV	Louder than heart sounds	No
Grade V	Louder than heart sounds	Yes
Grade VI	Very loud, can be appreciated with stethoscope just removed from contact with chest wall	Yes

2. Body Condition Score [7]

1	Bony prominences are easily visible. Poor muscle mass. No palpable fat layer.
2	Bony prominences are easily visible. Some loss of muscle mass. No palpable fat layer.
3	Vertical spinous processes of lumbar vertebra and pelvic wings are prominent. Ribs may be visible & are easily palpated. Obvious waist and abdominal tuck. Little fat palpable under skin
4	Ribs, spine and pelvis easily palpable. Marked waist and abdominal tuck. Minimal amount of fat cover.
5	Ribs, spine and pelvis palpable with mild amount of fat cover. Moderate waist and abdominal tuck.
6	Ribs, spine and pelvis palpable with moderate amount of fat cover. Waist and abdominal tuck are present but not prominent.
7	Ribs, spine and pelvis felt with pressure applied on palpation. Waist absent. Abdominal tuck may be present. Obvious fat layer under skin.
8	Ribs, spine and pelvis felt challenging to palpate. Waist absent, abdominal distension. Fat deposits over lumbar area and base of tail. Obvious fat layer under skin.
9	Ribs, spine and pelvis are not palpable. No waist. Abdominal distension and drooping. Large fat deposits over lumbar area, tail base, neck, limbs.

3. **Ultrasound Measurements**

A.

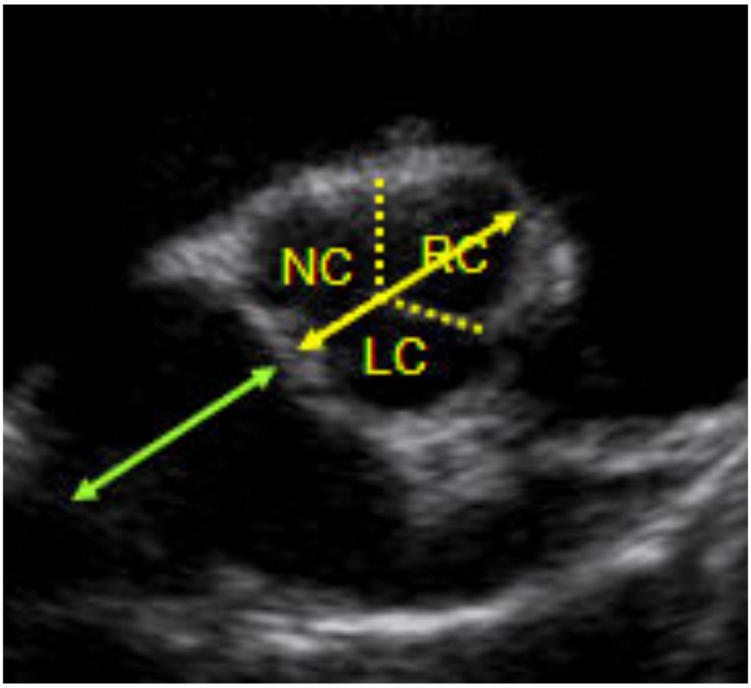

B.

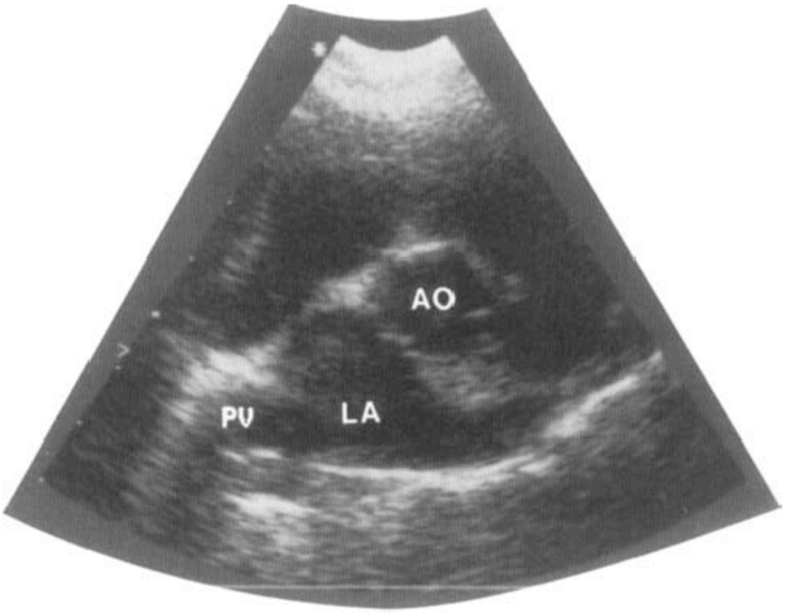

Figure: **A.** Right parasternal short-axis view showing the 2-D measurement technique. (arrows), and **B** dog with a normal **LA,** but where a pulmonary vein (PV) is entering the LA in the area of a desired measurement point.

4. Online Standard Data Capture Form

Login Page

If you have not registered yet, please register here

If you have already registered, please login below with your username and password:

**Username:**	
**Password:**	
Login	

Welcome Page

Welcome to the HAMLET website.

You can complete a new survey.

There are also 1 unsubmitted survey(s) from your centre. Select one to edit from the list below:

Case no. HAMLET0004 (12 Oct 2017): Lurcher 3 years, 2 months, FN

View the results of submitted surveys.

Data Collection Form

**Centre Name:**	Mitral Valve Clinic
**Case Number:**	HAMLET0027
**Date:**	25-01-2018
**Breed:**	CKCS
**Age:**	13 years 2 months
**Sex:**	○ F<br>○ M<br>○ FN<br>◉ MN
**Bodyweight:**	11.1 kilos
**Body Condition Score:**	4
**Heart rate (beats per minute):**	140
**Respiratory rate (breaths/min):**	
**Murmur Grade:**	IV
**Cough:**	○ Yes ◉ No
**Exercise tolerance (over past 6 months):**	○ Normal ◉ Decreased
**Predominant heart rhythm (on auscultation):**	○ Regular rhythm (sinus rhythm) ◉ Sinus arrhythmia ○ Other
**Appetite (over past 6 months):**	◉ Normal ○ Decreased
**Comments:**<br>None	
**Comorbitities:**<br>None	
**Medication the dog is currently receiving:**<br>(please give generic drug name, dose and indicate the length of time the dog has been receiving medication)<br>None	
**Echocardiographic heart size measurements**	
**LVIDD (cm):**	
**LA (cm):**	
**Ao (cm):**	
**If radiographs are taken please include the following -**	
**VHS:**	
Save	Submit

N.B. **Saving** the survey details will allow them to be added to or edited later. **Submitting** the results will close the survey and make the details available to the HAMLET researchers.

5. **Laboratory Forms**

IDEXX BioResearch	Request Form<br>BI/RVC HAMLET Study<br>Animal species: Dog	

**Patient Case Number:**	**HAMLET**<br>I__I__I__I__I	**Order ID:**<br><br>*Affix a sample barcode label here*
**Patient Forename:**<br>*In block capitals*		
**Owner Surname:**<br>*In block capitals*		
**Investigator Name:**<br>*In block capitals*		**Investigator Address:**
**Sampling Date**<br>_ _ / _ _ / 20_ _<br>DD MM YYYY		
**Lab-Number (for lab only):**		
**Material**<br>☐ Serum, white capped (1,0 ml)<br>☐ EDTA-Plasma (0,3ml)<br>☐ Serum, orange capped tube (0,5ml)		**Analyses (for lab only):**<br>H_S HAMLET (BR-AU1)<br>H_EP BNP<br>H_S LP
**Investigator Comments:**		
If sample dispatch was not the same day, please confirm that all samples were stored under frozen conditions (-20°C)<br>☐ Yes ☐ No ☐ N/A (sent on same day)		
**Laboratory acknowledgement of receipt:**<br><br>**Signature:**________________________ **Date:**______________________________		

Ludwigsburg Lab Contact: IDEXX BioResearch- Mörikestr. 28/3 - D- 71636 Ludwigsburg – Email to: IdexxBioresearch-Operations@idexx.com

IDEXX BioResearch	**Results Form**<br>**BI/RVC HAMLET Study**<br>**Animal species: Dog**	

Sampling Date	01.01.17	Order ID	454545454
Patient Forename	Charles	Laboratory No.	TE000932
Owner Surname	King	Veterinary Surgeon	Dr. W. Shakespeare
Case ID	HAMLET0001	Centre	The Hospital
Material Submitted	Serum x 2, EDTA plasma		

Test	Result	Sign	Reference value	Unit	Remark
**Study Profile HAMLET**					
Total protein	**60**		54 - 76	g/l	
Albumin	**40**		28 - 43	g/l	
Urea (BUN)	**2.1**	-	3.2 - 10.3	mmol/l	
Creatinine	**530**	+	< 124	umol/l	
Chloride	**6**	-	106 - 120	mmol/l	
Sodium	**6**	-	142 - 153	mmol/l	
Potassium	**6.0**	+	3.9 - 5.8	mmol/l	
Calcium	**6.0**	+	2.1 - 2.9	mmol/l	
Inorganic Phosphate	**6.0**	+	0.9 - 1.7	mmol/l	
Bilirubin (total)	**102.6**	+	< 6.8	umol/l	
Alkaline phosphatase	**6**		< 147	U/l	
ALT (GPT)	**6**		< 122	U/l	
Cholesterol	**0.2**		< 10.3	mmol/l	
Cholesterol (fasting dogs with regular body weight): < 7.8 mmol/l					
GGT	**6**		< 13	U/l	
Globulin	**20**	-	24 - 43	g/l	
Glucose	**0.3**	-	3.2 - 7.0	mmol/l	
SDMA (EIA)	**6**		0 - 14	ug/dl	1)
Cardiopet proBNP (ELISA)	**2000**			pmol/l	2)
**Sample for storage**					

Note:

1)
SDMA is within the reference interval and creatinine is increased. This combination of results is uncommon. Hemolysis, if present, can result in decreased SDMA. SDMA and creatinine can both be affected by biologic and assay variability resulting in fluctuations around the upper end of the reference interval; this can be seen with well-managed stable CKD and results will likely align as disease progresses. Creatinine can exceed the reference interval in muscular dogs with normal kidney function. Creatinine can be artifactually increased postprandially. If kidney disease is still suspected, a complete urinalysis should be performed on all patients to evaluate for inappropriate specific gravity, proteinuria or other evidence of kidney disease.

6. Laboratory Instructions

RVC Royal Veterinary College University of London | HAMLET

Sample Preparation and Shipping Instructions

United Kingdom

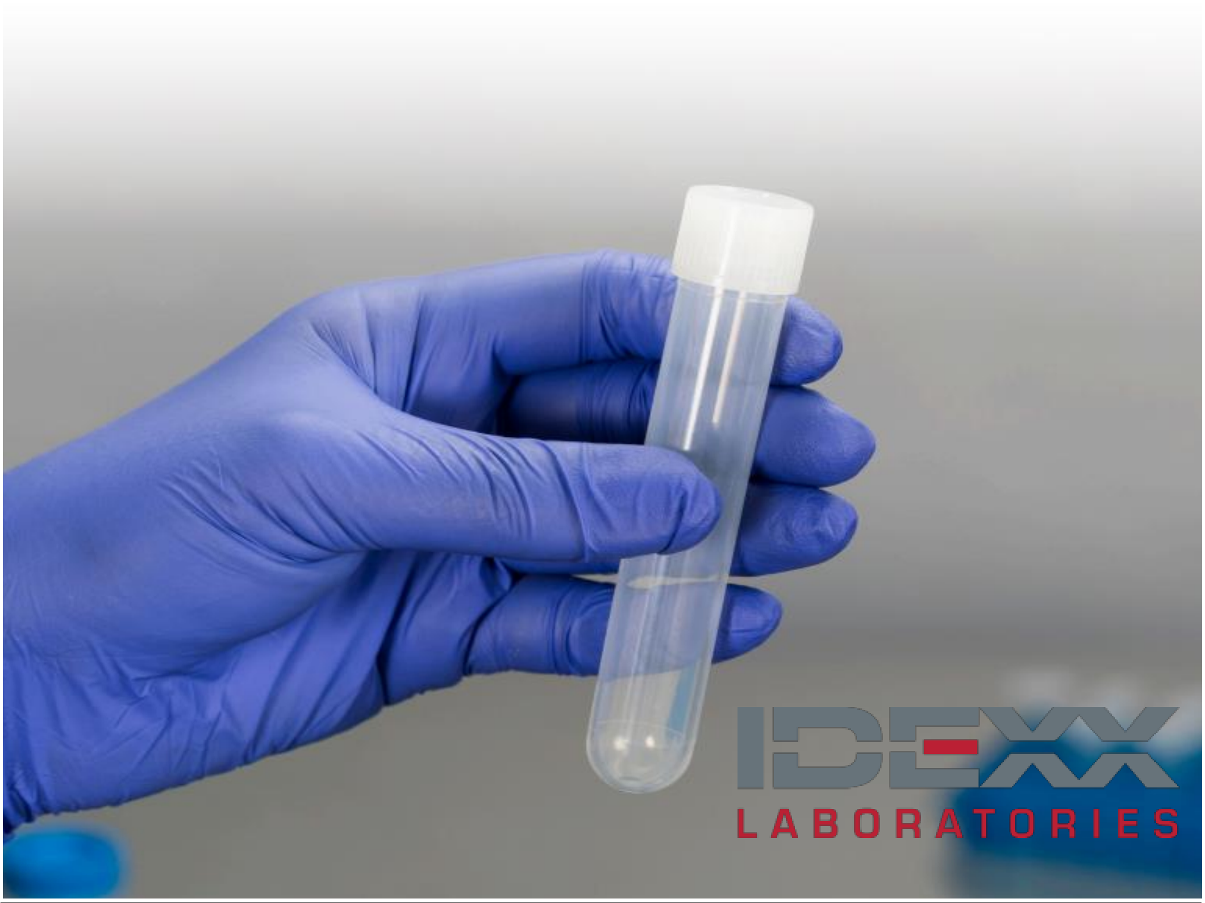

Sample Identification

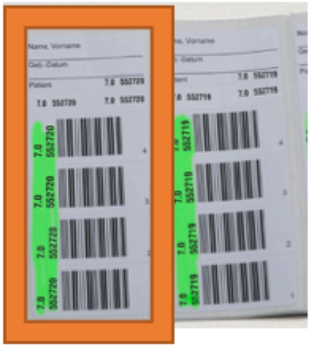

1. Please only use the green stickers for samples intended for the HAMLET study. Do not use these stickers for your routine samples.

2. Each column contains barcodes with the same number. Allocate one column per dog. Use the barcode stickers from the same column to label all of the allocated patient's samples and the corresponding request form.

3. Each column contains 1 identification sticker (at the top), 2 small stickers and 4 large stickers. The information on the small stickers is purely numerical. The large stickers contain an additional barcode.

Please label the white capped scaled serum tube with a large label; the EDTA plasma tube (smaller white capped tube, no scaling) with a small number label; and the orange capped serum storage tube with a small number label. This will be explained in further detail below.

4. Please complete the laboratory request form and place a large barcoded sticker (with the same number as the corresponding tubes) onto the form in the box labelled "Order ID".

Do not use tubes beyond their expiry date.
Do not take samples on Saturdays or Sundays.

Sample Preparation: Plasma

Requested whole blood volume: 1 ml
To produce a minimum volume of **0.3 ml plasma**

1. Collect the blood sample. You will need a total of **5mls** of blood from each patient.

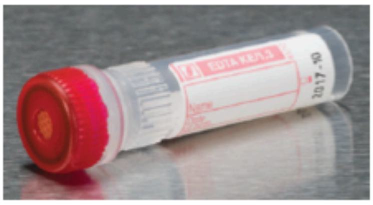

2. Fill one **red capped EDTA** tube with approximately **1 ml** of whole blood

3. Immediately invert the tube several times

4. Centrifuge the tube at low speed, about 1000g for 10 minutes

5. Carefully separate the plasma and place a minimum of **0.3 ml plasma** into the **small white capped tube** (no ring, not scaled)

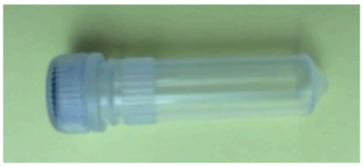

6. Place a **small** number sticker on this tube

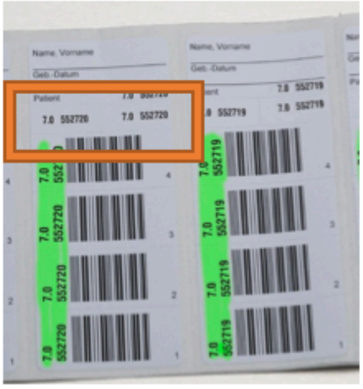

7. Dispose of the red capped EDTA tube

Sample Preparation: Serum

Requested whole blood volume: 4 ml
To produce two samples consisting of **1,0 ml** and **0,5 ml serum** per tube

1. Use the remaining **4 ml** (approx.) of whole blood to fill **3 – 4 white capped** tubes containing a ring.

2. To fill the tubes:

    - Fill up the tube with blood up to the 1.3 ml line.

    - Repeat until you have used all remaining sample. You should be able to fill 3 – 4 tubes.

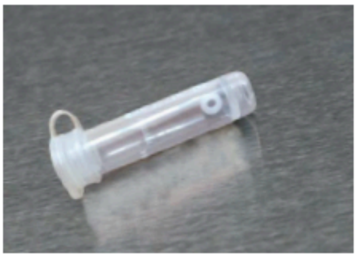

3. Allow the blood to coagulate fully (coagulation time varies between 30 min to 2 hours).

4. Once coagulated, centrifuge the tubes at a low speed: about 1000 g for 10 min.

5. Carefully separate the serum with a pipette and place it into two different tubes:

    - **1.5 ml** of serum in the **clear white capped** tube with the 1.0 - 2.5 ml scaling and the tapered bottom.
      Once filled, place a large barcode label on this tube.

    - **0.5 ml** of serum in the **orange capped** tube.
      Once filled, place a small label on the tube (the small label has no barcode, only numbers).

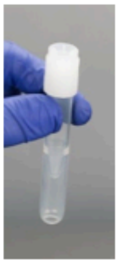

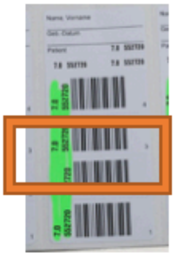

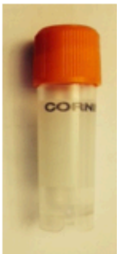

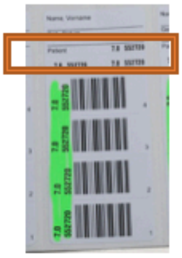

Sample Packaging & Delivery

1. Put the two serum sample tubes and the plasma sample tube in a clear specimen transport bag with an absorbent padding material and seal it carefully.

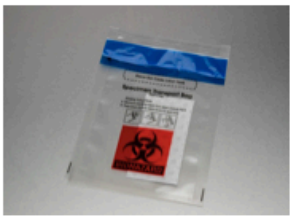

2. Place the sealed specimen bag, containing the serum and plasma samples, together with a frozen cooling pad in a Styrofoam box and close it. Put a rubber band around it to hold it shut.

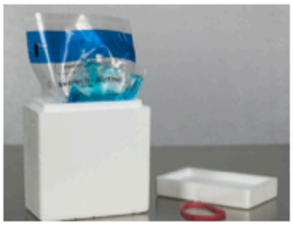

3. Place the closed Styrofoam box the into a silver isolation transport box

4. Stuff the transportation box with absorbent material to avoid rattling when the box is shaken

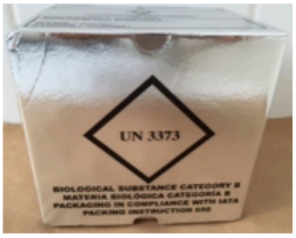

5. Add the completed submission form and seal the box. Place a DHL waybill sticker on the outside of the box (see below for more details). Avoid covering the address or dangerous goods label.

6. For quality reasons, it is essential to arrange the pickup for the same day as the sample collection occurs.

7. If the pickup cannot be arranged on the same day as the patient's examination, the samples should be stored at -20 °C (freezer temperature) and collected within 24 hours. On the day of collection proceed as described above.

Sample Delivery via DHL

The samples should be sent to IDEXX using a DHL courier

To order a DHL courier:

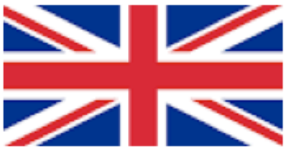

UK Investigators

Tel: **0844 248 08 44** Monday to Friday 7:30 - 22:00 h

IDEXX DHL client number: 968069022

Using DHL Waybills

1. Waybills ensure that the parcel is sent to IDEXX, cover courier costs and act as a proof of postage
2. Each waybill set contains a "voucher" sticker and an "archive" sticker
3. Place the "voucher" sticker on the outside of the silver transport box
4. Give the "archive" sticker to the DHL courier upon collection
5. In your pack, you will also find a form listing the codes of your waybills. The courier is required to sign this form upon collection of your samples. This can be used for tracking and as proof of postage. The courier should sign next to the number that corresponds with the code on "voucher" and "archive" waybills.

7. Manual Copy of Data Collection Form

Please complete in block capitals. Indicate correct responses by circling the appropriate option. Send by email to hamlet@rvc.ac.uk or by fax to 0207 468 5460

Centre Name	
Case Number (Use HAMLET followed by the date here DD/MM/YYYY)	
Date	
Breed	
Age	Years Months
Sex	F FN M MN
Bodyweight	Units
Body Condition Score	1 2 3 4 5 6 7 8 9
Heart Rate	Beats per minute
Respiratory Rate	Breaths per minute
Murmur Grade	I II III IV V VI
Cough	Yes No
Exercise Tolerance (over past 6 months)	Normal Decreased
Predominant Heart Rhythm<br>(if other, please indicate rhythm in comments box)	Regular rhythm (presumed sinus rhythm)<br>Sinus arrhythmia<br>Other
Appetite (over past 6 months)	Normal Decreased
Comments	
Comorbidities	
Medication the Dog is Currently Receiving<br>*Please give the generic drug name, dose and indicate the length of time the dog has been receiving medication)*	
LVIDD (cm)	
LA (cm)	
Ao (cm)	
If radiographs have been obtained:<br>VHS	

8/ Consent Form

Led by a team at the Royal Veterinary College, London, the HAMLET Study is investigating pre-clinical mitral valve disease in dogs and aims to improve the future management of this condition.

You can participate in the study by consenting to share some of the results from your dog's cardiac examination & blood tests.

Owner Name: ……………………………………… Patient Name: ………………………………………

I give permission for my dog to contribute data to the HAMLET study. I understand that the results from my dog's clinical history, physical examination, ultrasound scan and blood tests will be submitted for inclusion in this research project. I am aware that the primary investigators at the Royal Veterinary College will receive a copy of my dog's blood test results that will contain a copy of my dog's forename and my surname. I have been given the opportunity to ask questions and receive answers to my satisfaction.

I have read the information provided on this form and give consent for my dog to be enrolled in the HAMLET Study.

Signature: ………………………………………. Date: ……………………………………………….

Two copies of this Consent Form should be completed – one to be retained by the owner and the other to be retained by the Investigators
